# A Lamin Family-Based Signature Predicts Prognosis and Immunotherapy Response in Hepatocellular Carcinoma

**DOI:** 10.1155/2022/4983532

**Published:** 2022-11-10

**Authors:** Yongyu Yang, Wang Xiao, Ruoqi Liu, Lei Gao, Junzhang Chen, Heping Kan

**Affiliations:** ^1^Division of Hepatobiliopancreatic Surgery, Department of General Surgery, Nanfang Hospital, Southern Medical University, Guangzhou, Guangdong, China; ^2^Department of Neurosurgery, Nanfang Hospital, Southern Medical University, Guangzhou, Guangdong, China

## Abstract

**Background:**

Lamin family members play crucial roles in promoting oncogenesis and cancer development. The values of lamin family in predicting prognosis and immunotherapy response remain largely unclarified. Our research is aimed at comprehensively estimating the clinical significance of lamin family in hepatocellular carcinoma and constructing a novel lamin family-based signature to predict prognosis and guide the precise immunotherapy.

**Methods:**

The expression features and prognostic value of LMNA, LMNB1, and LMNB2 were explored in the TCGA and GEO databases. The biological functions of LMNB1 and LMNB2 were validated by in vitro assays. A lamin family-based signature was built using the TCGA training set. The TCGA test set, entire TCGA set, and GSE14520 set were used to validate its predictive power. Univariate and multivariate analyses were performed to evaluate the independence of the lamin family-based signature from other clinicopathological characteristics. A nomogram was constructed using the lamin family-based signature and TNM stage. The associations of this signature with molecular pathways, clinical characteristics, immune cell infiltration, and immunotherapy response were analyzed.

**Results:**

Lamin family members were upregulated in HCC. Upregulation of LMNB1 and LMNB2 promoted HCC proliferation, migration, and invasion. The predictive signature was initially established based on LMNB1 and LMNB2 which could effectively identify differences in overall survival, immune cell infiltration, and clinicopathological characteristics of high- and low-risk patients. The nomogram showed high prognostic predictive accuracy. Importantly, the lamin family-based signature was correlated with immune suppression and expression of immune checkpoint molecules.

**Conclusions:**

The lamin family-based signature is a robust biomarker to predict overall survival and immunotherapy response in HCC. High-risk score patients have a poorer overall survival and might be more sensitive to immunotherapy. This signature may contribute to improving individualized prognosis prediction and precision immunotherapy for HCC patients.

## 1. Introduction

Hepatocellular carcinoma (HCC) is the sixth most common cause of cancer-related death [1]. One of the characteristics of HCC is the lack of obvious clinical manifestations in the early stage. In addition, HCC is a highly malignant disease and is prone to invasion of blood vessels, postoperative recurrence, and metastasis [2]. Although various treatments are available, the prognosis of HCC patients remains unfavorable. BCLC and TNM staging is commonly used for the prognostic prediction of liver cancer. However, researchers have reported variable clinical outcomes in patients with the same BCLC stage and TNM stage [3, 4]. The above findings suggest that BCLC and TNM stage provide insufficient prognostic information for HCC patients. Immunotherapy has been recommended for advanced HCC [5, 6]. Unfortunately, durable responses to immunotherapy only occur in a portion of HCC patients [7, 8]. Hence, the discovery of precise biomarkers is warranted to improve the prognosis and prediction of the immunotherapy response of HCC.

Lamins form the main structure of the nuclear lamina and provide functional and structural links among the nucleoskeleton, genome, and cytoskeleton [9]. The nuclear lamina is essential in allowing cells to adequately respond to endogenous and exogenous stimuli, such as mechanical, chemical, and inflammatory stimuli [10]. The lamin family mainly contains three genes: LMNA, LMNB1, and LMNB2 [11]. They are involved in DNA replication and repair, tissue development, senescence, cellular proliferation, and gene expression regulation [12]. Recent studies have found that ectopic expression and/or localization of lamins are related to human cancer aggressiveness and metastasis. For example, LMNA expression was downregulated in colon cancer and was related to increased disease recurrence [13]. In Ewing sarcoma (EWS), high LMNA expression significantly inhibited the malignant behaviors of EWS and reduced YAP/TAZ nuclear recruitment by rescuing the LINC complex organization [14]. LMNB1 was upregulated in HCC, and upregulation of LMNB1 indicated adverse clinicopathological features, such as AJCC stage and number of lymph nodes [15]. Knockdown of LMNB1 was functionally relevant for melanoma and lung adenocarcinoma (LUAD) progression by influencing cell senescence and DNA damage [16, 17]. Upregulation of LMNB2 expression promoted cancer cell proliferation and indicated worse disease-free survival in colorectal cancer and bladder cancer [18, 19]. Moreover, lamin family genes play functional roles in the immune system. LMNA could significantly augment T helper 1 cells differentiation [20]. LMNB1 is closely correlated with T helper 2 cell infiltration [21].

Collectively, the previous findings have identified that lamin family members have important roles in tumor development and the immune microenvironment. A better understanding of the lamin family could provide new insight for the management of human tumors. However, a whole picture of the lamin family from the aspects of prognosis and immunotherapy response has not been systematically investigated. Therefore, we aimed to comprehensively explore clinical and immunological characteristics of the entire lamin family in HCC and establish a lamin family-based signature, thus improving prognosis prediction and precision immunotherapy for HCC.

In this study, by performing comprehensive multidataset bioinformatics analyses and fundamental experiments, we established a lamin family-based signature and validated its accuracy and reliability both internally and externally. We established a nomogram to quantitatively guide the personalized therapy of HCC. Finally, we identified the values of this signature in the prognosis and immunotherapy response prediction. As a result, we found that the lamin family-based signature could contribute to improving individualized prognosis prediction and precise immunotherapy.

## 2. Materials and Methods

### 2.1. Data Collection and Preprocessing

**Figure 1:**
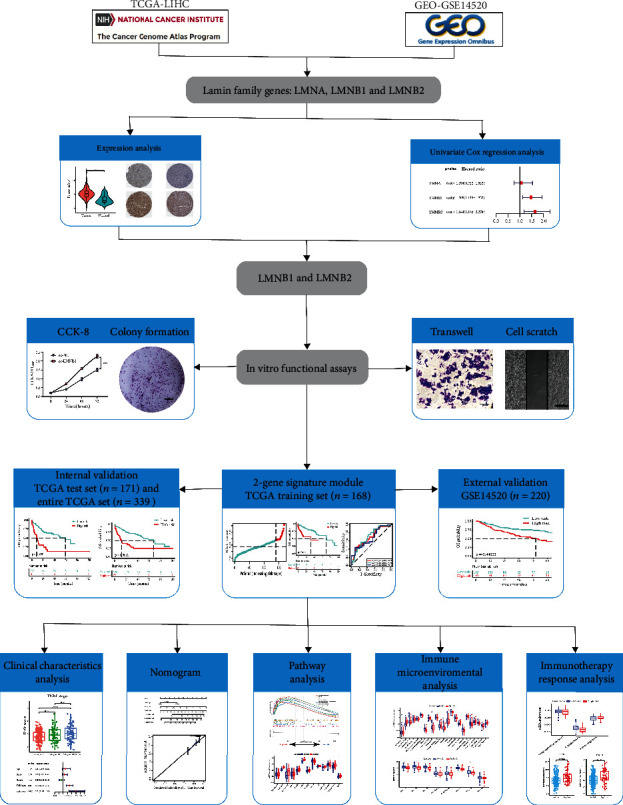
Flow chart of this study.

The flow chart of this study is shown in Figure 1. The RNA sequencing profiles and clinical data of HCC patients were downloaded from The Cancer Genome Atlas (TCGA) (https://portal.gdc.cancer.gov) and the Gene Expression Omnibus (GEO) database (http://www.ncbi.nlm.nih.gov/geo). The genetic count data from TCGA were normalized and log2 transformed. The clinical information and processed microarray expression files of HCC patients in the GSE14520 dataset were downloaded from the GEO database. HCC samples lacking complete clinical information were removed. If a gene had more than one probe, the mean expression value was chosen as its expression value. Finally, the data of 339 patients from TCGA and 220 patients from GSE14520 were extracted for subsequent analyses (Table 1). Because the data in the GEO and TCGA databases are publicly available, we performed the current study following the access policies and publication guidelines of the above databases. Therefore, no ethical approval or informed consent was required for this study.

### 2.2. Expression Analysis of Lamin Family

We explored the mRNA and protein expression levels of all lamin family members. First, we analyzed the difference in mRNA expression levels between HCC and normal tissues using the TCGA transcriptomic data. Then, we analyzed the protein levels of LMNA, LMNB1, and LMNB2 from the Human Protein Atlas (HPA; http://www.proteinatlas.org).

### 2.3. Evaluation of the Prognostic Significance of Lamin Family Members

To systematically evaluate the association between all lamin family genes and the overall survival (OS) of HCC patients, we randomly divided 339 HCC patients in the TCGA dataset into a training set (*n* = 168) and a test set (*n* = 171) using the “caret” R package. All lamin family members, including LMNA, LMNB1, and LMNB2, were used to conduct univariate Cox regression analyses in the above datasets as well as the GSE14520 set.

### 2.4. Cell Culture and Plasmid Transfection

The normal liver cell line (LO2, GNHu 6) and liver cancer cell lines (Huh7, SCSP-526; MHCC97H, TCHu 94; HepG2, SCSP-510; Hep3B, SCSP-5045) were purchased from the Chinese Academy of Sciences (Shanghai, China) and were cultured in DMEM with 10% fetal bovine serum (FBS). All cells were incubated at 37°C with 5% CO_2_. The pcDNA3.1-LMNB1 and pcDNA3.1-LMNB2 plasmids were generated by Umine Biotechnology (Guangzhou, China, Uminebio-20211223). Cell transfection was performed using Lipofectamine 2000 reagent (Life Technology) according to the manufacturer's instructions. Western blotting was performed to assess the overexpression efficiency.

### 2.5. Western Blotting

Western blotting was performed by following the previous description [22]. The primary antibodies were as follows: LMNB1 (Santa Cruz, sc-377000), LMNB2 (Santa Cruz, sc-377379), and GAPDH (Abcam, ab8245). The protein band was visualized using an enhanced chemiluminescence kit (ECL, Millipore) in an electrochemiluminescence imaging analysis system.

### 2.6. Cell Counting Kit-8 (CCK-8) Assay

After transfection for 48 h, Huh7 and MHCC97H HCC cells were reseeded in 96-well plates (2 × 10^3^ cells per well). Ten microliters of CCK-8 solution (Solarbio, CA1210) were added to each well. The absorption at 450 nm was measured with a microplate reader.

### 2.7. Colony Formation Assay

The transfected HCC cells were seeded in 6-well plates (1 × 10^3^ cells per well) and cultured for two weeks. Subsequently, the cells were stained with 0.1% crystal violet solution. Finally, visible colonies with more than 50 cells were counted.

### 2.8. Cell Scratch Assay

The migratory ability of the Huh7 and MHCC97H cell lines was evaluated by scratch assay. Transfected HCC cells were seeded in 6-well plates. After the cells reached 90% confluence, a wound was created by a 100 *μ*L pipette tip. Wound closure areas were recorded at 0 h and 24 h under an inverted microscope.

### 2.9. Transwell Invasion Assay

A total of 5 × 10^4^ HCC cells transfected with plasmids were seeded in the upper chamber of a Matrigel-coated chamber (8 *μ*m pore size; Corning) and cultured in serum-free DMEM. After 24 h, the cells with good invasive ability were traversed to the lower surface of the membrane. Then, 4% paraformaldehyde was used to fix the invasive cells, and 0.1% crystal violet was used to stain the invasive cells. Finally, the invasive cells were counted under an inverted microscope.

### 2.10. Construction of a Lamin Family-Based Signature

According to the above analyses, LMNB1 and LMNB2 are OS-related lamin family members. Therefore, LMNB1 and LMNB2 were used to construct a risk score (RS) predictive signature. The RS was calculated using the following formula: RS = coefficient of LMNB1∗expression of LMNB1 + coefficient of LMNB2∗expression of LMNB2. After the RS was calculated for each HCC patient, an optimal cutoff value was determined by the “surv_cutpoint” function in the “survminer” R package. Then, the patients were divided into low- and high-risk groups according to the above optimal cutoff value. The reliability of this RS was assessed by Kaplan-Meier survival analysis and log-rank tests.

### 2.11. Validating the Lamin Family-Based Signature

The performance of the signature was validated by the TCGA test set, entire TCGA set, and GSE14520 set. HCC patients in the above datasets were divided into two groups according to the optimal cutoff value. The difference on OS was evaluated by the Kaplan-Meier method. Moreover, the relationship between the RS and clinicopathological characteristics was analyzed. Univariate and multivariate Cox regression analyses were performed to estimate the independent predictive performance of the RS.

### 2.12. Construction and Evaluation of a Nomogram

To further quantitatively evaluate the OS, we constructed a nomogram on the basis of all independent prognostic factors using the “rms” R package. Calibration plots were used to evaluate its predictive power. ROC curve analysis was performed to compare the predictive performance of the nomogram and TNM stage.

### 2.13. Functional and Pathway Analyses

To reveal the potential biological functions and signaling pathways, we conducted gene set enrichment analysis (GSEA) [23] using the “clusterProfiler” R package. In addition, the correlation between RS and oncogenic hallmarks was estimated. We collected 13 common oncogenic hallmark signatures (Table S1) from previous studies [24, 25] and calculated the enrichment scores of HCC patients using single-sample gene set enrichment analysis (ssGSEA) [26].

### 2.14. Immune Cell Infiltration Analysis

We further estimated characteristics of immune cell infiltration using ssGSEA. The marker gene sets (Table S2) were collected from previous studies [27]. Enrichment scores were calculated to represent the immune cell infiltration abundance.

### 2.15. Prediction of the Immunotherapy Response

The predictive performance of the risk score on immunotherapy response was estimated with immune-related pathways and the expression levels of immune checkpoint inhibitory molecules. Immune-related pathway enrichment scores were calculated based on the previous immune signatures (Table S3) [25, 28]. The immune checkpoint inhibitory molecules used in this study included CTLA4, HAVCR2, LAG3, PDCD1, and TIGIT.

### 2.16. Statistical Analysis

Data analyses were performed using R software (version 4.0.1) and IBM SPSS software, version 22.0. For comparisons between two groups, the Wilcoxon test and Student's *t*-test were employed. Kruskal-Wallis test was used for comparisons of more than two groups. Kaplan-Meier survival analysis and the log-rank test were used to assess the prognostic differences in different groups. Correlations between RS groups and clinicopathologic features were analyzed with the chi-squared test. A *P* value <0.05 was considered statistically significant, ^∗^*P* < 0.05, ^∗∗^*P* < 0.01, and ^∗∗∗^*P* < 0.001.

## 3. Results

### 3.1. Aberrant Expression of Lamin Family Members

**Figure 2:**
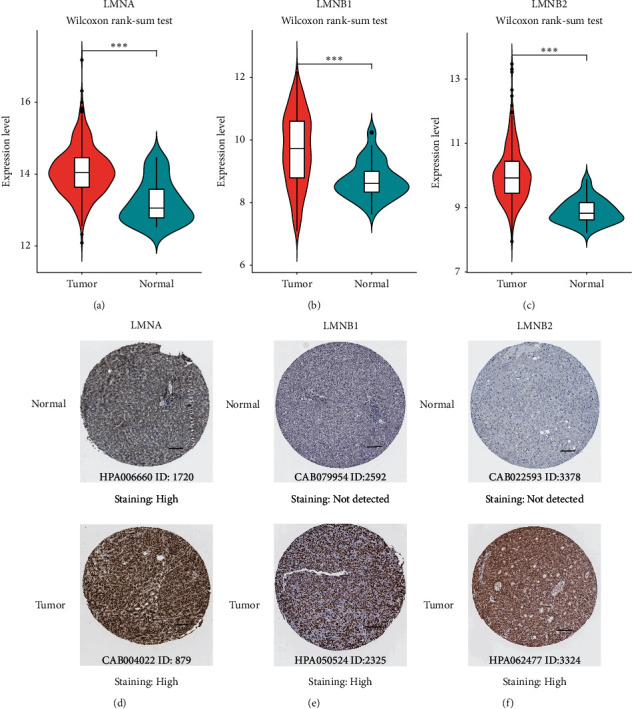
Expression levels of lamin family members in HCC. (a–c) The mRNA expression levels of LMNA, LMNB1, and LMNB2 in the TCGA database. Wilcoxon rank-sum test, ^∗∗∗^*P* < 0.001. (d–f) Representative immunohistochemistry images of LMNA, LMNB1, and LMNB2 in HCC and normal tissues from the HPA database; scale bar = 100 *μ*m.

The expression patterns of lamin family members were analyzed from the TCGA and the HPA public databases. As shown in Figure 2, the mRNA expression levels of LMNA (Figure 2(a)), LMNB1 (Figure 2(b)), and LMNB2 (Figure 2(c)) were significantly increased in liver tumor tissues (all *P* < 0.001). From the HPA database, high LMNA staining in HCC and normal tissues was observed. However, a stronger LMNA staining intensity was found in HCC tissues (Figure 2(d)). In addition, higher protein levels of LMNB1 and LMNB2 (Figures 2(e)–2(f)) were observed in HCC. In normal tissues, LMNB1 and LMNB2 proteins were not detected. But high staining intensity of LMNB1 and LMNB2 protein was detected in HCC tissues. These results suggested that both the transcriptional and protein levels of the lamin family members were high expressed in HCC.

### 3.2. Prognostic Significance of Lamin Family Members

**Figure 3:**
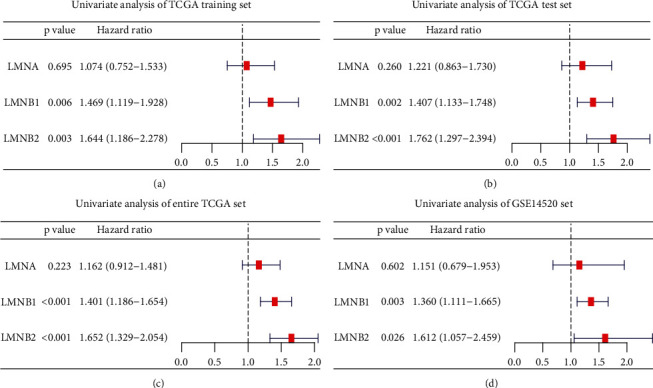
Univariate Cox regression analyses of the lamin family members in HCC datasets. (a) TCGA training set. (b) TCGA test set. (c) Entire TCGA set. (d) GSE14520 set.

We further explored the prognostic value of lamin family members. The results of univariate analyses showed that LMNB1 and LMNB2 were unfavorable prognostic factors in the TCGA training set (LMNB1: *P* = 0.006, HR = 1.469 (1.119-1.928); LMNB2: *P* = 0.003, HR = 1.644 (1.186-2.278); Figure 3(a)), TCGA test set (LMNB1: *P* = 0.002, HR = 1.407 (1.133-1.748); LMNB2: *P* < 0.001, HR = 1.762 (1.297-2.394); Figure 3(b)), entire TCGA set (LMNB1: *P* < 0.001, HR = 1.401 (1.186-1.654); LMNB2: *P* < 0.001, HR = 1.652 (1.329-2.054); Figure 3(c)), and GSE14520 set (LMNB1: *P* = 0.003, HR = 1.360 (1.111-1.665); LMNB2: *P* = 0.026, HR = 1.612 (1.057-2.549); Figure 3(d)). However, LMNA showed no impact on OS in the above four datasets.

### 3.3. Functional Validation of LMNB1 and LMNB2 in HCC

**Figure 4:**
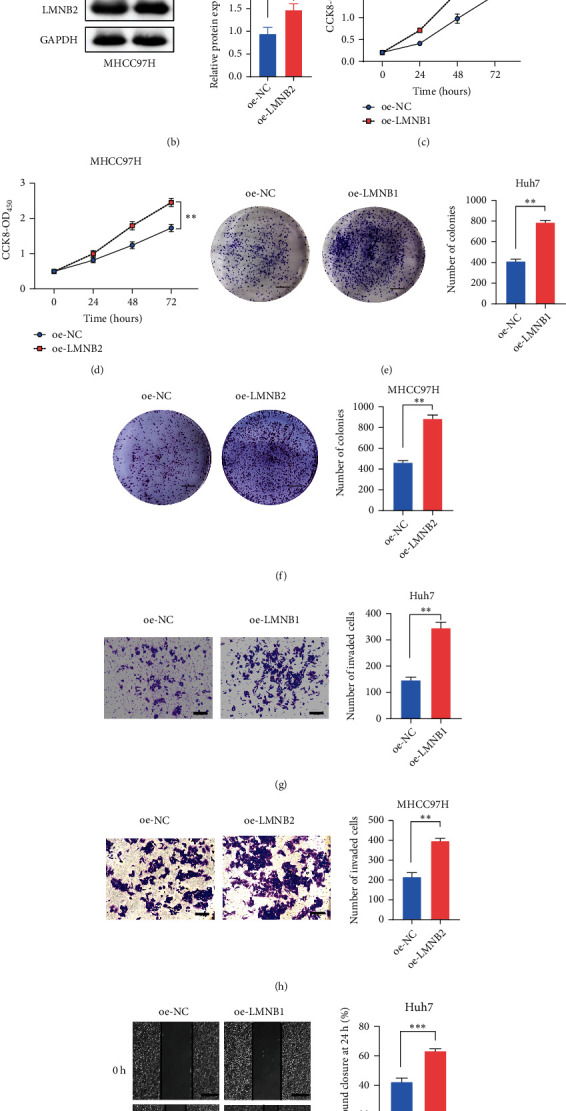
Functional validation of LMNB1 and LMNB2 in HCC. (a) The protein expression levels of LMNB1 and LMNB2 in normal liver cells and four HCC cell lines. (b) Western blotting analyses for overexpression efficacy detection in Huh7 and MHCC97H cells after transfection with LMNB1 and LMNB2 plasmids. (c, d) CCK-8 assays of Huh7 and MHCC97H cells with LMNB1 and LMNB2 overexpression, respectively. (e, f) Colony formation assays of Huh7 and MHCC97H cells with LMNB1 and LMNB2 overexpression, respectively; scale bar = 5 mm. (g, h) Transwell invasion assays of Huh7 and MHCC97H cells with LMNB1 and LMNB2 overexpression, respectively; scale bar = 100 *μ*m. (i, j) Cell scratch assays of Huh7 and MHCC97H cells with LMNB1 and LMNB2 overexpression, respectively; scale bar = 500 *μ*m. Data are presented as the mean ± SD. Statistical differences between two groups were evaluated using Student's *t*-test, ^∗^*P* < 0.05, ^∗∗^*P* < 0.01, and ^∗∗∗^*P* < 0.001.

LMNB1 and LMNB2 were highly expressed in HCC and affected patients' OS. We speculated that LMNB1 and LMNB2 play biological roles in HCC. Therefore, we explored the biological functions of LMNB1 and LMNB2 through in vitro assays. First, we detected the protein levels of LMNB1 and LMNB2 in HCC and normal liver cell lines. The results showed that the expression of LMNB1 and LMNB2 protein was higher in HCC cell lines (Figure 4(a)). In addition, LMNB1 had a low expression level in Huh7 cells, while LMNB2 had a low expression level in MHCC97H cells. Therefore, we overexpressed LMNB1 and LMNB2 in Huh7 and MHCC97H cells, respectively. Western blotting results validated that the protein levels of LMNB1 and LMNB2 in HCC cells were significantly upregulated after transfection with the corresponding plasmids (Figure 4(b)). Then, functional assays were performed. The CCK-8 and colony formation assays showed that LMNB1 and LMNB2 upregulation significantly enhanced the proliferation (Figures 4(c) and 4(d) and colony formation (Figures 4(e) and 4(f) abilities of HCC cells (all *P* < 0.05). Transwell invasion and cell scratch assays showed that LMNB1 and LMNB2 upregulation significantly enhanced cell invasion (Figures 4(g) and 4(h) and migration (Figures 4(i) and 4(j) in HCC (all *P* < 0.05). Overall, LMNB1 and LMNB2 promote HCC malignancy.

For the sake of exploring the underling mechanism of LMNB1 and LMNB2 in HCC, we divided HCC patients into two groups: LMNB1 high- and low-expression and LMNB2 high- and low-expression groups. Differently expressed genes (DEGs) between these two groups were screened using the “limma” R package. The threshold value for selecting DEGs was set as a false discovery rate (FDR) < 0.05 and log2(Fold Change) > 1. Then, the DEGs were used for Gene Ontology (GO) and Kyoto Encyclopedia of Genes and Genomes (KEGG) analyses via the Database for Annotation, Visualization, and Integrated Discovery (DAVID), version 6.8 (https://david.ncifcrf.gov/home.jsp). As a result, 1379 DEGs were identified between HCC samples with LMNB1 high and low expression. GO biological functions of these DEGs were mainly enriched in cell differentiation, transcription factor activity, cell division, mitotic cell cycle, and sequence-specific DNA binding (Figure S1(a)). KEGG pathways associated with these DEGs were mainly enriched in chemical carcinogenesis-receptor activation, Rap1 signaling pathway, p53 signaling pathway, cellular senescence, Fanconi anemia pathway, and calcium signaling pathway (Figure S1(b)). In addition, 2023 DEGs were identified between HCC samples with LMNB2 high and low expression. GO biological functions of these DEGs were mainly enriched in cell differentiation, cell adhesion, extracellular matrix organization, growth factor activity, sequence-specific DNA binding, and positive regulation of cell proliferation (Figure S1(c)). KEGG pathway analysis of these DEGs was mainly enriched in PI3K-Akt signaling pathway, cAMP signaling pathway, neuroactive ligand-receptor interaction, cytokine-cytokine receptor interaction, IL-17 signaling pathway, and Hippo signaling pathway (Figure S1(d)). These functions and pathways are closely-associated cancer development, indicating that LMNB1 and LMNB2 could affect HCC progression through multiple cellular functions and signaling pathways. However, further fundamental experiments are needed to illustrate how LMNB1 and LMNB2 regulate these signaling pathways.

### 3.4. Construction and Verification of the Lamin Family-Based Signature

**Figure 5:**
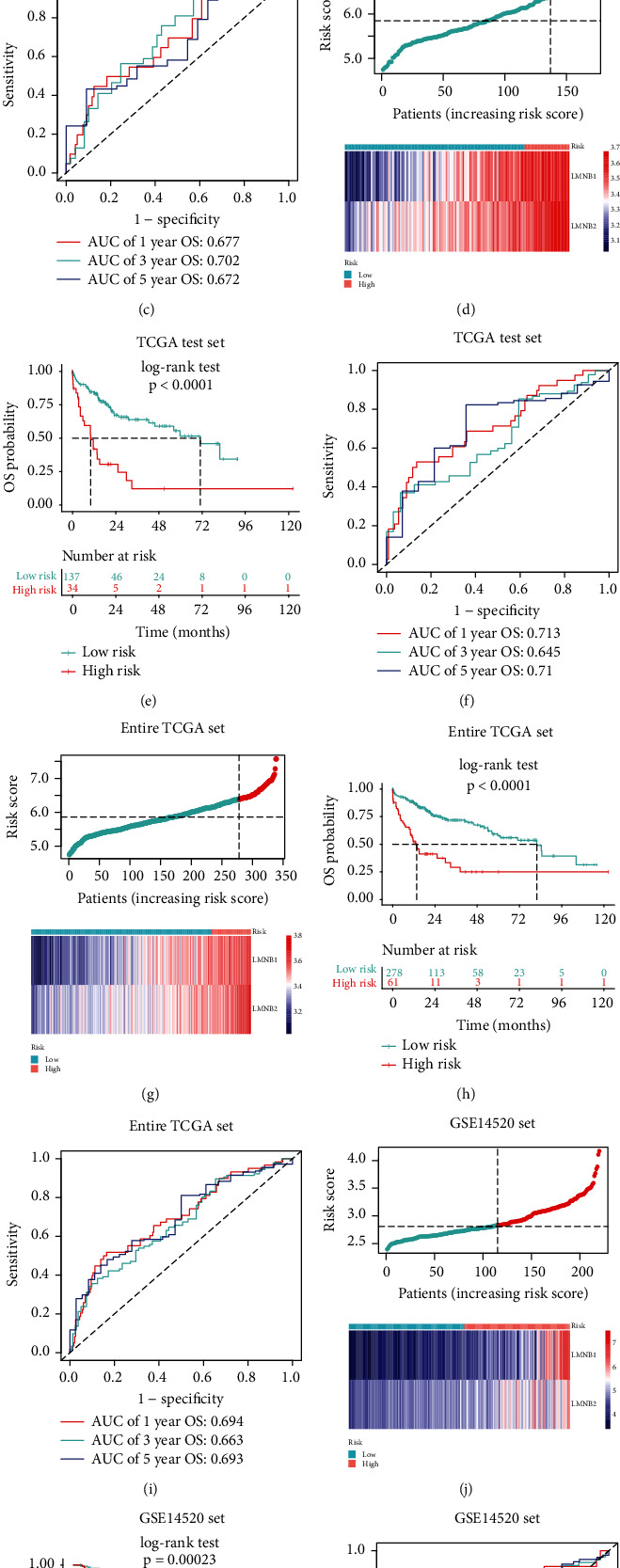
Construction and evaluation of the lamin family-based signature. (a, d, g, j) Risk score distribution of LMNB1 and LMNB2 in patients with different risk scores in the TCGA training set (a), TCGA test set (d), entire TCGA set (g), and GSE14520 set (j). (b, e, h, k) Kaplan-Meier survival analysis for HCC patients in the TCGA training set (b), TCGA test set (e), entire TCGA set (h), and GSE14520 set (k); log-rank test, all *P* < 0.0001. (c, f, i, l). ROC curve analyses of the 1-year, 3-year, and 5-year overall survival of the lamin family-based signature from the TCGA training set (AUC = 0.667, 0.702, 0.672, respectively) (c), TCGA test set (AUC = 0.713, 0.645, 0.71, respectively) (f), entire TCGA set (AUC = 0.694, 0.663, 0.693, respectively) (i), and GSE14520 set (AUC = 0.6, 0.62, 0.669, respectively) (l).

Because the expression of LMNB1 and LMNB2 was independently associated with the OS, we selected LMNB1 and LMNB2 to construct a predictive signature. The risk score (RS) of this signature was calculated as follows: RS = expression of LMNB1^∗^ 0.2609 + expression of LMNB2^∗^ 0.3369. To evaluate the reliability of this RS, we divided 168 HCC patients into low- (*n* = 140) and high- (*n* = 28) risk two groups in the TCGA training set based on the optimal cutoff value (Figure 5(a)). Then, Kaplan-Meier analysis results showed that high-risk patients had a worse OS than the low-risk patients (Figure 5(b), log-rank test, *P* = 4*e* − 04). The areas under the curves (AUCs) for the 1-year, 3-year, and 5-year OS of the RS were 0.677, 0.702, and 0.672, respectively (Figure 5(c)). For internal validation, 171 HCC patients in the TCGA test set were stratified into the low- (*n* = 137) and high- (*n* = 34) risk groups based on the same optimal cutoff value (Figure 5(d)). Similar to the TCGA training set, high-risk patients were significantly associated with poorer OS (Figure 5(e), log-rank test, *P* < 0.0001). AUCs for 1-year, 3-year, and 5-year overall survival of the RS in the TCGA test set were 0.713, 0.645, and 0.710, respectively (Figure 5(f)). In addition, 339 HCC patients in the entire TCGA set were stratified into the low- (*n* = 278) and high- (*n* = 61) risk groups too (Figure 5(g)). High-risk patients were also associated with poorer prognosis (Figure 5(h), log-rank test, *P* < 0.0001). The AUCs for 1-year, 3-year, and 5-year overall survival of the RS in entire TCGA set were 0.694, 0.663, and 0.693, respectively (Figure 5(i)). For the external validation, we divided 220 HCC patients into low-risk (*n* = 115) and high-risk (*n* = 105) groups in the GSE14520 set based on the risk score (Figure 5(j)). Consistent with the above results, high-risk patients had a worse OS (Figure 5(k), log-rank test, *P* = 0.00023). AUCs of 1-year, 3-year, and 5-year overall survival of the RS in the GSE14520 set were 0.600, 0.620, and 0.669, respectively (Figure 5(l)). These findings revealed the robustness of the RS in predicting OS of HCC.

### 3.5. Clinical Evaluation of the RS Model

**Figure 6:**
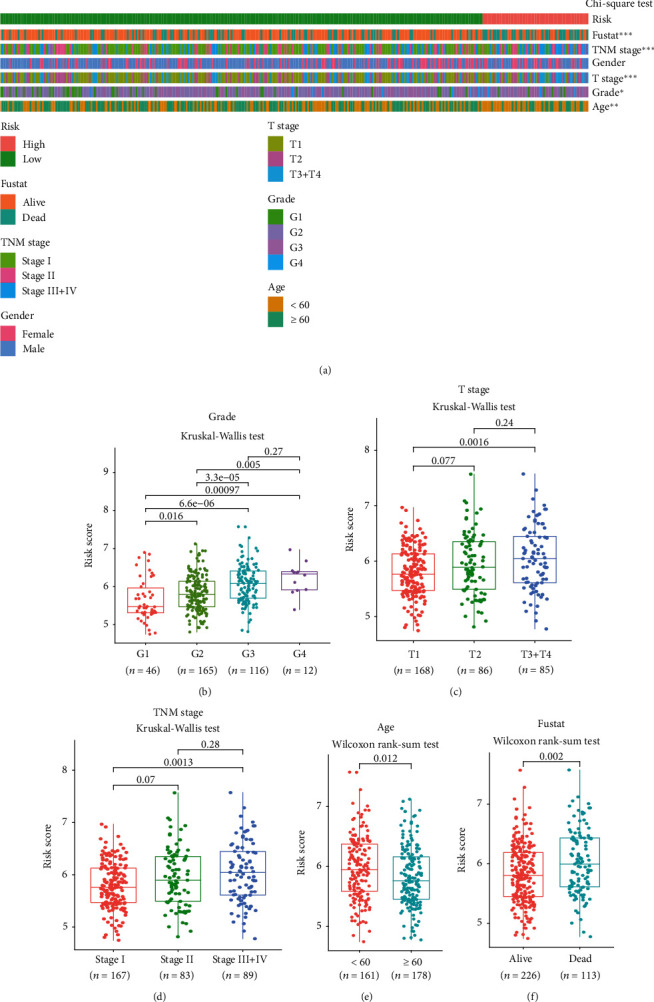
Associations between the RS and clinicopathological characteristics. (a) Strip chart of the clinicopathological characteristics in HCC patients; chi-squared test, ^∗^*P* < 0.05, ^∗∗^*P* < 0.01, and ^∗∗∗^*P* < 0.001. (b–d) Association between risk score and grade (b), T stage (c), and TNM stage (d); Kruskal-Wallis test, *P* values indicated. (e) Association between risk score and age; Wilcoxon rank-sum test, *P* = 0.012. (f) Association between risk score and survival status; Wilcoxon rank-sum test, *P* = 0.002.

We analyzed the correlations between the RS and clinical characteristics in the TCGA cohort (*n* = 339). As Figure 6 shows, the RS was associated with survival status (*P* < 0.001), TNM stage (*P* < 0.001), T stage (*P* < 0.001), grade (*P* < 0.05), and age (*P* < 0.01) in the strip chart (Figure 6(a)). In addition, the box line scatter diagrams showed that high-risk patients significantly exhibited advanced grade (Figure 6(b)), T stage (Figure 6(c)), and TNM stage (Figure 6(d)), as well as lower age (Figure 6(e)), and a high frequency of death (Figure 6(f)). These findings suggested that high-risk patients often had adverse clinical characteristics.

**Figure 7:**
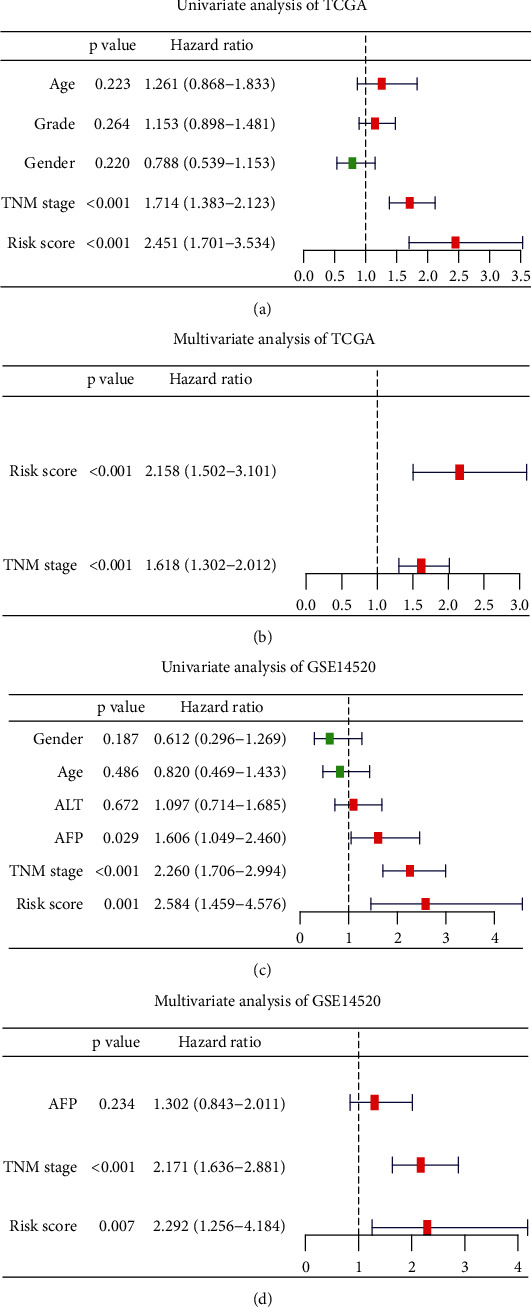
Independent prognostic value of the RS in HCC. (a–b) Univariate Cox analyses of RS and clinical characteristics in the TCGA cohort (*n* = 339) (a) and GSE14520 (*n* = 220), (b). (c) Multivariate Cox analyses of RS and TNM stage in the TCGA cohort. (d) Multivariate Cox analyses of RS, AFP, and TNM stage in GSE14520.

To prove the independence of the RS, we included the RS, age, grade, gender, and TNM stage in univariate analyses in the TCGA cohort (*n* = 339). The results suggested that TNM stage (HR = 1.714 (1.383-2.123), *P* < 0.001) and RS (HR = 2.451 (1.701-3.534), *P* < 0.001) were risk factors for OS (Figure 7(a)). Multivariate analysis further confirmed that TNM stage (HR = 1.168 (1.302-2.012), *P* < 0.001) and the RS (HR = 2.158 (1.502-3.101), *P* < 0.001) were independently correlated with patients' OS (Figure 7(b)). Consistent with the TCGA cohort, univariate analyses in the GSE14520 set (*n* = 220) revealed that TNM stage (HR = 2.260 (1.706-2.994), *P* < 0.001) and RS (HR = 2.584 (1.459-4.576), *P* = 0.001) were closely associated with OS (Figure 7(c)). Multivariate analysis also revealed that TNM stage (HR = 2.171 (1.636-2.881), *P* < 0.001) and RS (HR = 2.292 (1.256-4.184), *P* < 0.007) were independent risk factors of HCC (Figure 7(d)). Therefore, the lamin family-based signature risk score model is a robust biomarker for OS prediction.

### 3.6. Construction and Evaluation of the Nomogram

**Figure 8:**
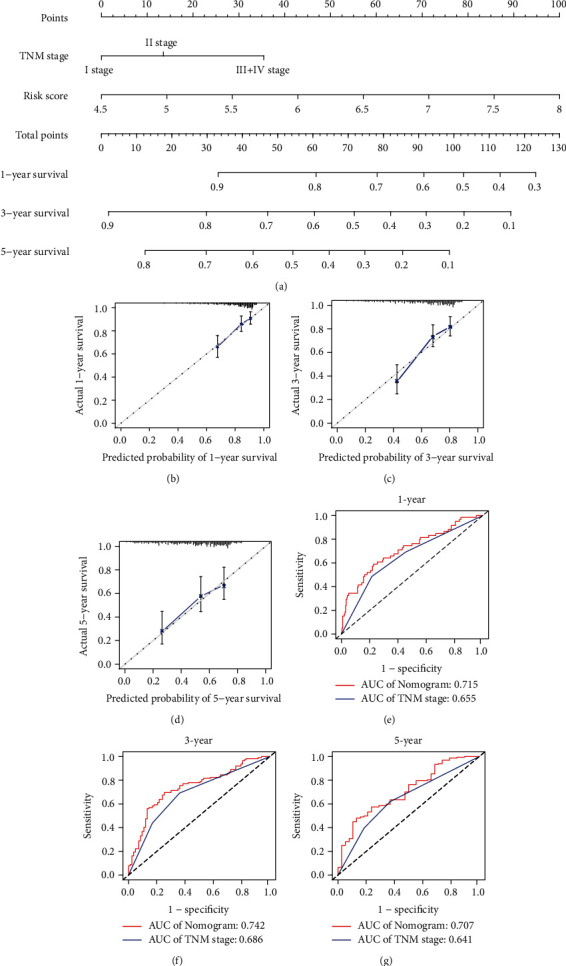
Construction and evaluation of the nomogram for predicting the OS of HCC patients. (a) Nomogram for predicting 1 year, 3 years, and 5 years OS. (b–d) Calibration plots of the nomogram for predicting OS at 1 year (b), 3 years (c), and 5 years (d). (e–g) ROC curves of the nomogram and TNM stage at 1 year (AUC = 0.715, 0.655, respectively, e), 3 years (AUC = 0.742, 0.686, respectively, f), and 5 years (AUC = 0.707, 0.641, respectively, g).

We used the RS and TNM stage to construct a nomogram to quantitatively predict OS at 1 year, 3 years, and 5 years (Figure 8(a)). The calibration curve and area under the curve (AUC) were employed to validate the performance of the nomogram. The calibration curves showed that the nomogram-predicted OS at 1 year (Figure 8(b)), 3 years (Figure 8(c)), and 5 years (Figure 8(d)) was approximately the same as the actual values. In addition, the AUCs of the nomogram were larger than those of the TNM stage. The AUCs of the nomogram at 1 year (Figure 8(e)), 3 years (Figure 8(f)), and 5 years (Figure 8(g)) were 0.715, 0.742, and 0.707, respectively. These results suggested that the nomogram has a favorable predictive accuracy.

### 3.7. Functional and Pathway Analyses

**Figure 9:**
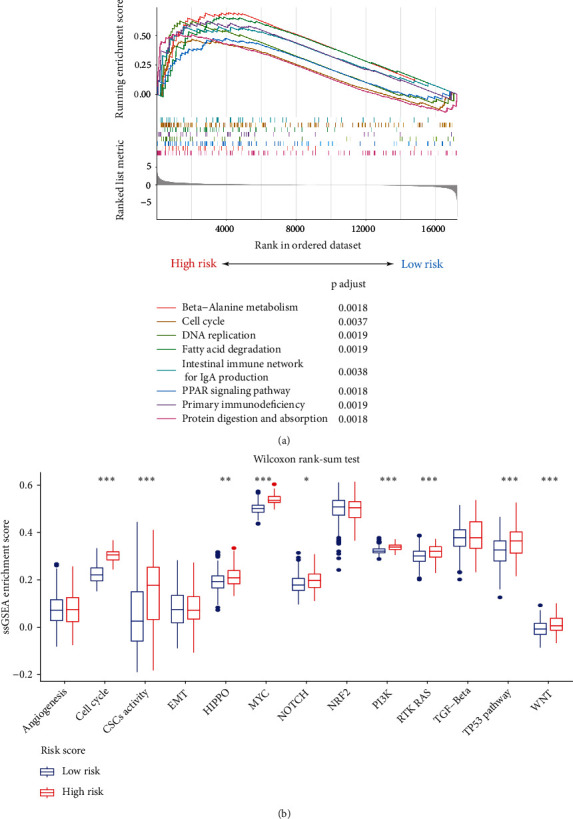
Functional and pathway analyses. (a) Enriched functions and pathways associated with RS. (b) Correlations between oncogenic pathways and the RS; Wilcoxon rank-sum test, ^∗^*P* < 0.05, ^∗∗^*P* < 0.01, and ^∗∗∗^*P* < 0.001.

GSEA was employed to investigate the differences in cancer-related biological behaviors and pathways between the high- and low-risk groups. The results suggested that beta-alanine metabolism, the intestinal immune network for IgA production, fatty acid degradation, the PPAR signaling pathway, primary immunodeficiency, the cell cycle, DNA replication, and protein digestion and absorption were significantly enriched in the high-risk group (Figure 9(a)). We further explored the association between RS and oncogenic pathways through ssGSEA. As shown in Figure 9(b), high activity levels of tumor-associated pathways, including the cell cycle, Hippo, MYC, NOTCH, RAS, TP53, WNT, PI3K, and cancer stem cells (CSCs), were observed in the high-risk group. Most of these pathways play important roles in promoting HCC development [29–31]. These findings indicated that the RS has the potential to reflect pathway-based molecular characteristics of HCC.

### 3.8. Immune Cell Infiltration Landscape in Different Risk Score HCC Patients

**Figure 10:**
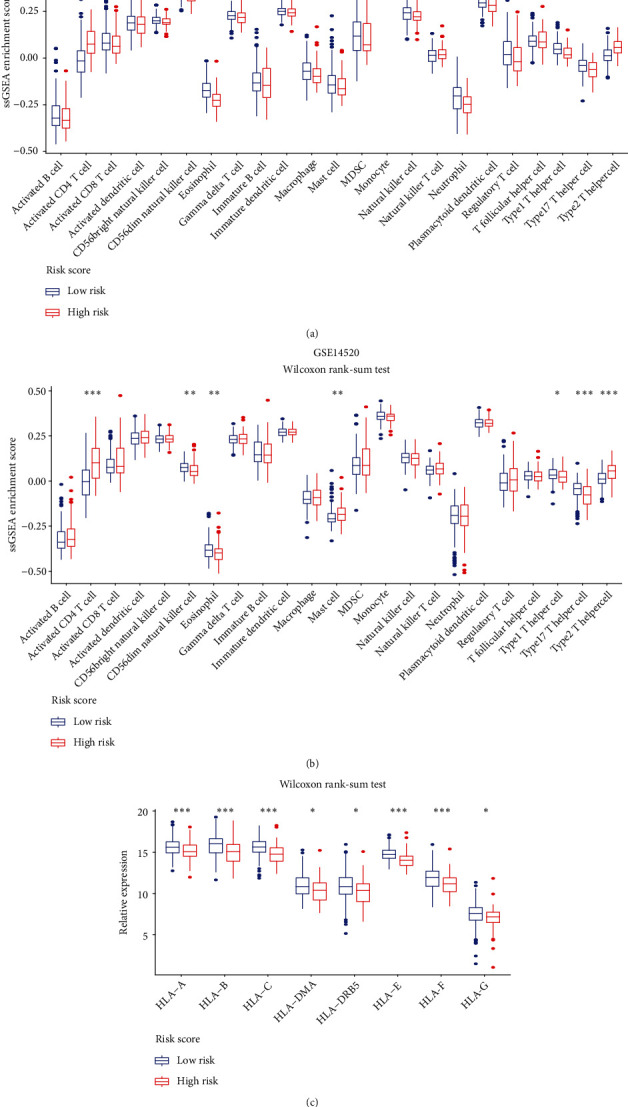
Immune landscape analysis. (a, b) Relative enrichment scores of immune cell infiltration in the TCGA-LIHC dataset (a) and GSE14520 dataset (b). (c) Differential expression of HLA genes in the high- and low-risk groups. Wilcoxon rank-sum test, ^∗^*P* < 0.05, ^∗∗^*P* < 0.01, and ^∗∗∗^*P* < 0.001.

The ssGSEA enrichment score of 23 immune cell types was calculated to estimate the relative cell infiltrating abundance in the tumor microenvironment. As shown in Figures 10(a) and 10(b), the high-risk patients exhibited a significantly decreased abundance of CD56dim natural killer cell, eosinophil, monocyte, natural killer cell, plasmacytoid dendritic cell, macrophage, gamma delta T cell, type 1 helper cell, type 17 helper cell, and type 2 T helper cell. In addition, expression analysis revealed that many human leukocyte antigen (HLA) genes were downregulated in the high-risk group (Figure 10(c)). The above findings indicated that high-risk patients have an immunosuppressive phenotype. Restoring the immune system of high-risk patients could effectively fight against tumor.

### 3.9. The Lamin Family-Based Signature Predicts Immunotherapy Responses

**Figure 11:**
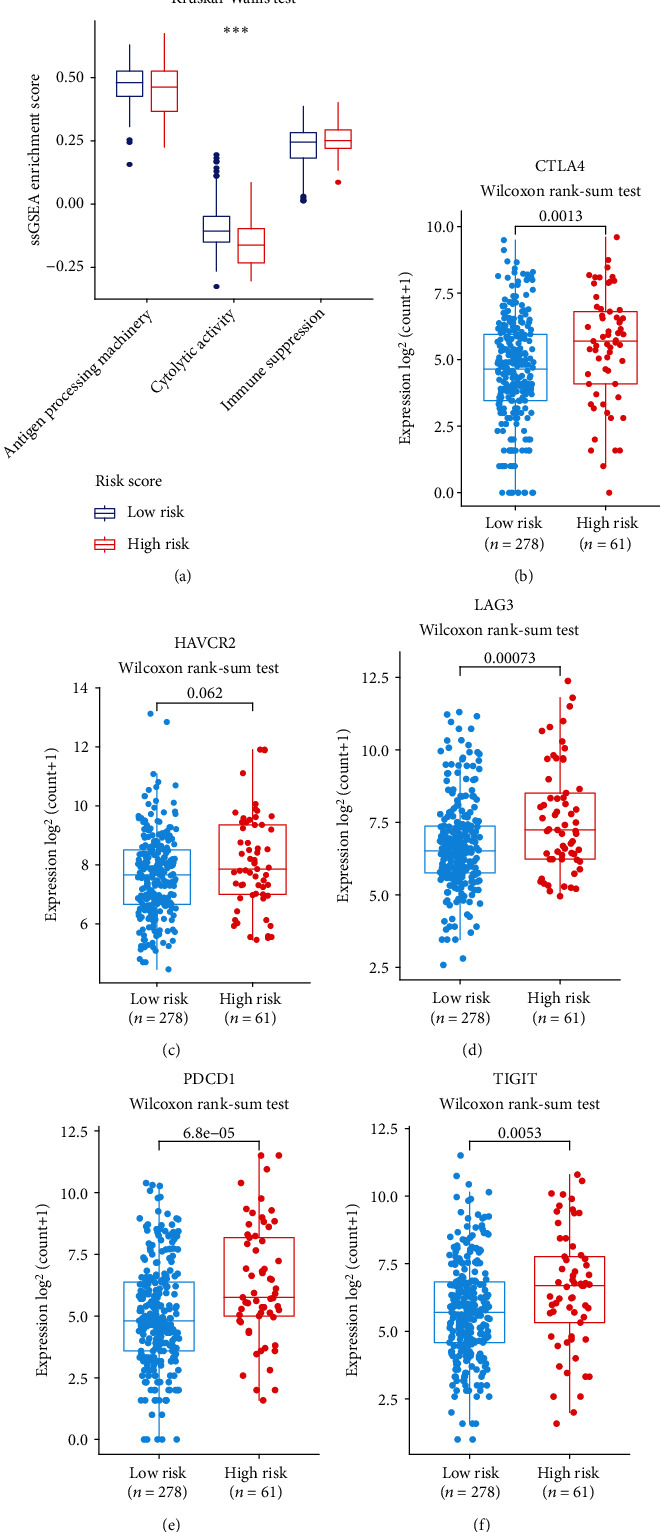
Immunotherapy response analysis. (a) Correlations between immune-related pathways and RS; Kruskal-Wallis test, ^∗∗∗^*P* < 0.001. (b–f) Correlations between several prominent immune checkpoints and RS: CTLA4 (b), HAVCR2 (c), LAG3 (d), PDCD1 (e), and TIGIT (f); Wilcoxon rank-sum test, *P* values indicated.

Patient immune status and immune checkpoint molecules are common biomarkers for judging immunotherapy responses. Through GSEA, a significant correlation between the RS and immune system was observed. A high-risk score seems to indicate an immunosuppressive phenotype. To further confirm this hypothesis, we calculated the ssGSEA enrichment scores of immune-related pathways and analyzed associations between RS and patient immune suppression, cytolytic activity, and antigen processing machinery. In the high-risk group, lower cytolytic activity was observed (Figure 11(a)), which indicating a higher immune suppression status. Correlation analysis suggested that high-risk HCC patients presented higher levels of CTLA-4 (Figure 11(b)), HAVCR2 (Figure 11(c)), LAG3 (Figure 11(d)), PDCD1 (Figure 11(e)), and TIGIT (Figure 11(f)) than low-risk HCC patients. These findings indicate that high-risk patients are more likely to benefit from immunotherapy.

## 4. Discussion

The development of genomics and bioinformatics tools has enabled researchers to identify reliable biomarkers and construct robust gene family-based predictive signatures for cancer prognosis prediction. For example, Zhang et al. established a high-precision prognostic model for predicting the clinical outcomes of lung adenocarcinoma (LUAD) patients based on TNF family genes [32]. Wang et al. incorporated six CXCL family genes into a glioma prognostic model [33]. These gene family-based signatures showed excellent prognostic predictive value for the OS of patients. In HCC, TNM stage and BCLC stage have been used to classify patients into several groups. These staging methods have failed to provide individual prognostic prediction for patients [34, 35]. Recent studies have reported the significant influence of lamin family members on tumorigenesis, cancer development, and cancer metastasis [36–38]. However, the value of all family members in predicting the prognosis and immunotherapy response of HCC is largely unknown. We hypothesized that the lamin family has potent prognostic value and predictive value for the immunotherapy response in HCC patients.

Our research found that lamin family genes were highly expressed in HCC. Prognostic analysis suggested that LMNB1 and LMNB2 are risk factors for unfavorable clinical outcomes. LMNB1 and LMNB2 are the fundamental structures of the nucleoskeleton. LMNB1 is an oncogene in various cancers. In HCC, our previous study demonstrated that silencing of LMNB1 inhibited HCC proliferation and metastasis both in vitro and in vivo [39]. Another study reported that circulating LMNB1 is a diagnostic biomarker for early stage liver cancer [15]. In lung adenocarcinoma, silencing of LMNB1 significantly inhibited cell proliferation capacity and cell motility [40]. Elevated expression of LMNB2 was associated with tumor immune cell infiltrates and poor OS in HCC [41]. In addition, LMNB2 was significantly upregulated in colorectal cancer. LMNB2 upregulation could promote colorectal cancer progression by regulating p21 expression [18]. These previous findings were consistent with our study, emphasizing that LMNB1 and LMNB2 play important roles in tumor progression.

Individual lamin family genes could affect HCC progression and predict prognosis for HCC patients. We speculated that the combination of lamin family genes could improve the prognostic prediction and management of HCC patients. Thus, an OS prediction signature was constructed based on LMNB1 and LMNB2. The predictive capacity of this signature was both internally and externally validated. Notably, the lamin family-based signature exhibited a favorable predictive accuracy and could successfully discriminate clinical outcomes. Moreover, RS was closely associated with tumor grade, survival status, T stage, age, and TNM stage (all *P* < 0.05). These results demonstrated that high-risk HCC patients are more likely to have unfavorable clinicopathological characteristics. After evaluating the independence of the risk score, we constructed a nomogram to quantitatively guide individualized treatment of HCC patients. Calibration and ROC curves showed that the predictive ability of the nomogram was satisfactory. Our study comprehensively demonstrated the prognostic significance of the lamin family-based signature in HCC. We reported that this lamin family-based signature has the potential to predict the prognosis of HCC.

HCC is a heterogeneous disease with a complex genetic background. We explored the underlying mechanism causing different clinical outcomes between patients with different risk scores. GSEA revealed that the functions related to digestion, absorption, immunity, and the cell cycle were enriched in high-risk patients. Correlation analysis demonstrated that high-risk patients have high activation in the MYC, PI3K, RAS, WNT, and CSC signaling pathways (all *P* < 0.05). The importance of the above signaling pathways in promoting tumor development has been identified. MYC strongly promotes HCC progression by activating WDR4 transcription [42]. PI3Ks belong to the heterodimeric lipid kinase family, and alterations in PI3K pathway genes are involved in multiple cancers [43]. Liu et al. reported that downregulation of ARHGAP20 significantly inhibited HCC development by moderating the PI3K-AKT pathway [44]. The RAS pathway plays a fundamental role in moderating cellular processes and has been implicated in HCC malignant phenotypes [45]. The WNT pathway plays pivotal roles in tumor metastasis [46]. CSCs are a type of cell with a self-renewal capacity that will differentiate into different cancer cells under a specified condition [47]. Researchers have extensively studied CSCs due to their strong relationships with cancer relapse. The above findings suggested that the high-risk population has greater predisposition for tumorigenesis and recurrence. More medical strategies should be developed to detect or prevent tumor recurrence in patients with high-risk scores.

In the present study, patients with high-risk scores had lower levels of immune cell infiltration, indicating that these patients had an immunosuppressive status. Currently, immunotherapy has greatly improved the clinical outcomes of HCC by enhancing the immune system. Targeting of immune checkpoints is a major method of immunotherapy. Therefore, immune checkpoints are routinely used to judge whether patients are suitable for immunotherapy [48]. Further, analysis found that high-risk patients tended to exhibit increased expression levels of PDCD1 (*P* = 6.8*e* − 05), CTLA-4 (*P* = 0.0013), LAG3 (*P* = 0.00073), and TIGIT (*P* = 0.0053). The above immune checkpoints are coinhibitory receptors. High expression of these coinhibitory receptors indicates a dysfunctional or exhausted phenotype of T cells and negative regulation of the antitumor response [49]. Strong evidence has shown that these coinhibitory receptors exhibit unprecedented efficacy in the treatment of some cancers [50]. Since the RS strongly correlated with these immune checkpoints, the lamin family-based signature could serve as a powerful tool that helps us to determine who is sensitive to immunotherapy.

Our study highlights the lamin family-based signature as a predictive biomarker of overall survival and immunotherapy response for HCC. This signature was sufficiently validated through comprehensive evaluation of multiple datasets, indicating its strength and reliability. Currently, precision medicine is asking us to accurately evaluate the prognosis of and provide individualized treatment for patients with cancers. The signature is an independent prognostic factor, and the combination of the lamin family-based signature and TNM stage could quantitatively predict overall survival for patients with HCC. Therefore, this signature could help to improve individualized treatment and long-term prognosis for patients with HCC. In addition, the present dilemma of immunotherapy is the lack of robust biomarkers to recognize patients who are suitable for immunotherapy. Another merit of the lamin family-based signature is its close association with the immune microenvironment activation, especially immune cell infiltration, HLA gene expression, and immune checkpoint molecular expression. This association indicates that the lamin family-based signature has the potential to enhance precision immunotherapy; that is, we could judge whether a patient is suitable for immunotherapy according to the risk score. However, this study has some limitations. First, systematic bias is inevitable because the data were downloaded from public databases. Second, clinical trials are needed to further externally validate the lamin family-based signature, because the externally validated dataset was from the GEO database, which lacks a good design in terms of this study. Third, further fundamental experiments are needed to explore the underlying mechanisms that LMNB1 and LMNB2 promote HCC progression. In addition, the direct interaction between the lamin family members and immune checkpoints needs to be further clarified using additional experiments.

In conclusion, our study demonstrated that lamin family members are highly expressed in HCC. LMNB1 and LMNB2 are prognostic predictors for HCC. The lamin family-based signature risk model is a prognostic biomarker and a robust predictive biomarker for the immunotherapy response of HCC. The risk score reflects immune cell infiltration in HCC and is associated with the expression of immune checkpoint molecules. Incorporating the risk score and TNM stage to a nomogram could quantitatively predict OS. Overall, this study may contribute to improving individualized prognosis prediction and precision immunotherapy for HCC patients.

## Figures and Tables

**Table 1:** Clinical information of HCC patients enrolled in this study.

Characteristics	TCGA	GSE14520
Number of patients	339	220
Age (years)		
(i) <60	161 (47.49%)	177 (80.45%)
(ii) ≥60	178 (52.50%)	43 (19.55%)
Gender		
(i) Male	231 (68.14%)	191 (86.81%)
(ii) Female	108 (31.86%)	29 (13.18%)
TNM stage		
(i) Stage I	167 (49.26%)	92 (41.82%)
(ii) Stage II	83 (24.48%)	78 (35.45%)
(iii) Stage III	84 (24.78%)	50 (22.73%)
(iv) Stage IV	5 (1.47%)	0 (0%)
Grade		
(i) G1	46 (13.57%)	—
(ii) G2	164 (48.38%)	—
(iii) G3	117 (34.51%)	—
(iv) G4	12 (3.54%)	—
ALT		
(i) ≤50 (U/L)	—	130 (59.09%)
(ii) >50 (U/L)	—	90 (40.91%)
AFP		
(iii) ≤300 (ng/ml)	—	121 (55%)
(iv) >300 (ng/ml)	—	99 (45%)
Survival status		
(i) Alive	226 (66.67%)	135 (61.36%)
(ii) Dead	113 (33.33%)	85 (38.64%)

## Data Availability

The public data included in this study was originated from The Gene Expression Omnibus (https://www.ncbi.nlm.nih.gov/geo/) and The Cancer Genome Atlas (https://portal.gdc.cancer. gov/). The experimental data that support the findings of this study are available on request from the corresponding author.

## Supplementary Materials

Supplementary 1 Figure S1: GO and KEGG analyses. Figure S1: (a) GO and (b) KEGG enrichment analyses between HCC samples with high and low LMNB1 expression. (c) GO and (d) KEGG enrichment analyses between HCC samples with high and low LMNB2 expression. Enrichment results indicated that LNMB1 and LNMB2 were closely associated with cancer-related cellular functions and signaling pathways. BP: biological process; CC: cellular component; MF: molecular function.

Supplementary 2 Table S1: Tumor hallmark-related signatures used in this study. Table S2: the cell type gene sets used for immune cell infiltration analysis. Table S3: immune signatures used in this study.
