# Supplementary figures and images for: A Lamin Family-Based Signature Predicts Prognosis and Immunotherapy Response in Hepatocellular Carcinoma
Source: J Immunol Res. 2022 Nov 10;2022:4983532. doi: 10.1155/2022/4983532 (PMC9673181; doi:10.1155/2022/4983532)

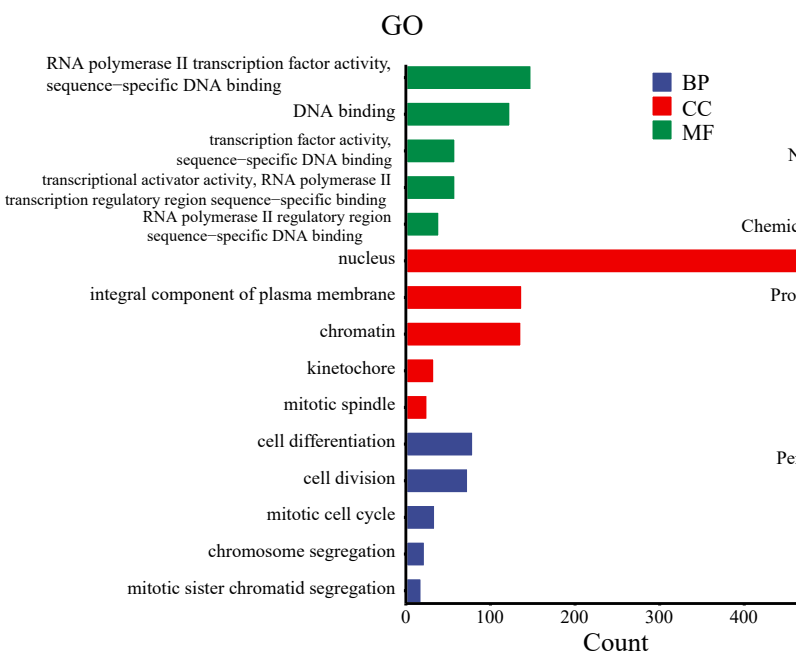

(a)

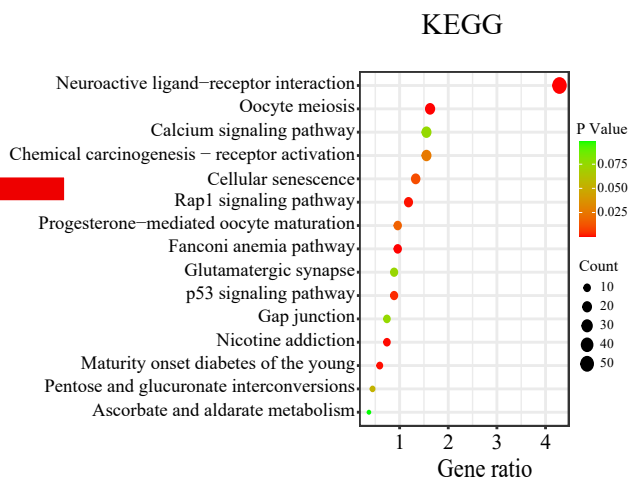

(b)

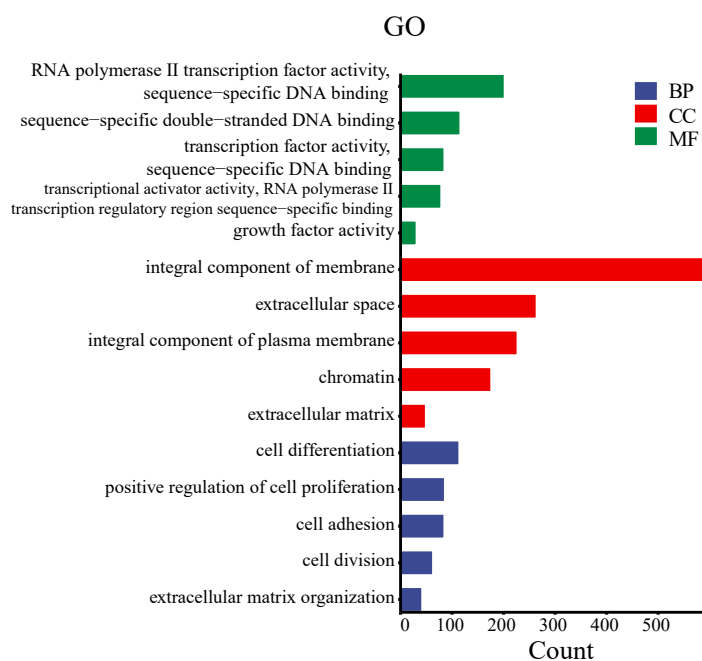

(c)

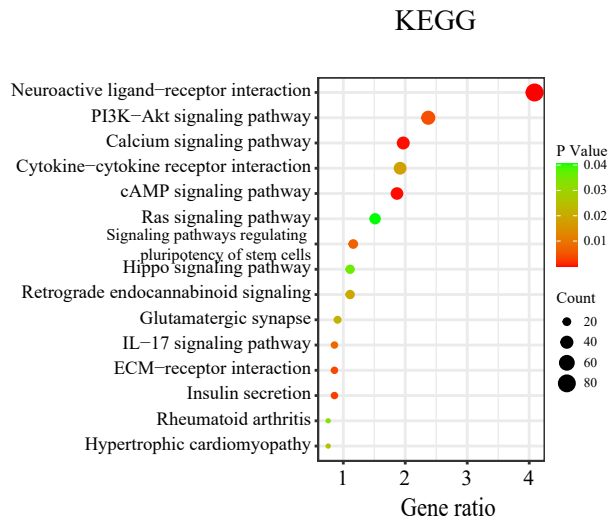

(d)

Supplement: Supplementary 1 — Figure S1: GO and KEGG analyses. Figure S1: (a) GO and (b) KEGG enrichment analyses between HCC samples with high and low LMNB1 expression. (c) GO and (d) KEGG enrichment analyses between HCC samples with high and low LMNB2 expression. Enrichment results indicated that LNMB1 and LNMB2 were closely associated with cancer-related cellular functions and signaling pathways. BP: biological process; CC: cellular component; MF: molecular function. [file 4983532.f1.pdf]
