# Supplementary material for: A Lamin Family-Based Signature Predicts Prognosis and Immunotherapy Response in Hepatocellular Carcinoma
Source: J Immunol Res. 2022 Nov 10;2022:4983532. doi: 10.1155/2022/4983532 (PMC9673181; doi:10.1155/2022/4983532)
Supplement: Supplementary 2 — Table S1: Tumor hallmark-related signatures used in this study. Table S2: the cell type gene sets used for immune cell infiltration analysis. Table S3: immune signatures used in this study. [file 4983532.f2.docx]

| **Table S1** Tumor hallmark-related signatures used in this study | |
| --- | --- |
| Metagene | Signatures |
| ABL1 | Cell cycle |
| ANAPC1 | Cell cycle |
| ANAPC10 | Cell cycle |
| ANAPC11 | Cell cycle |
| ANAPC13 | Cell cycle |
| ANAPC2 | Cell cycle |
| ANAPC4 | Cell cycle |
| ANAPC5 | Cell cycle |
| ANAPC7 | Cell cycle |
| ATM | Cell cycle |
| ATR | Cell cycle |
| BUB1 | Cell cycle |
| BUB1B | Cell cycle |
| BUB3 | Cell cycle |
| CCNA1 | Cell cycle |
| CCNA2 | Cell cycle |
| CCNB1 | Cell cycle |
| CCNB2 | Cell cycle |
| CCNB3 | Cell cycle |
| CCND1 | Cell cycle |
| CCND2 | Cell cycle |
| CCND3 | Cell cycle |
| CCNE1 | Cell cycle |
| CCNE2 | Cell cycle |
| CCNH | Cell cycle |
| CDC14A | Cell cycle |
| CDC14B | Cell cycle |
| CDC16 | Cell cycle |
| CDC20 | Cell cycle |
| CDC23 | Cell cycle |
| CDC25A | Cell cycle |
| CDC25B | Cell cycle |
| CDC25C | Cell cycle |
| CDC26 | Cell cycle |
| CDC27 | Cell cycle |
| CDC45 | Cell cycle |
| CDC6 | Cell cycle |
| CDC7 | Cell cycle |
| CDK1 | Cell cycle |
| CDK2 | Cell cycle |
| CDK4 | Cell cycle |
| CDK6 | Cell cycle |
| CDK7 | Cell cycle |
| CDKN1A | Cell cycle |
| CDKN1B | Cell cycle |
| CDKN1C | Cell cycle |
| CDKN2A | Cell cycle |
| CDKN2B | Cell cycle |
| CDKN2C | Cell cycle |
| CDKN2D | Cell cycle |
| CHEK1 | Cell cycle |
| CHEK2 | Cell cycle |
| CREBBP | Cell cycle |
| CUL1 | Cell cycle |
| DBF4 | Cell cycle |
| E2F1 | Cell cycle |
| E2F2 | Cell cycle |
| E2F3 | Cell cycle |
| E2F4 | Cell cycle |
| E2F5 | Cell cycle |
| EP300 | Cell cycle |
| ESPL1 | Cell cycle |
| FZR1 | Cell cycle |
| GADD45A | Cell cycle |
| GADD45B | Cell cycle |
| GADD45G | Cell cycle |
| GSK3B | Cell cycle |
| HDAC1 | Cell cycle |
| HDAC2 | Cell cycle |
| MAD1L1 | Cell cycle |
| MAD2L1 | Cell cycle |
| MAD2L2 | Cell cycle |
| MCM2 | Cell cycle |
| MCM3 | Cell cycle |
| MCM4 | Cell cycle |
| MCM5 | Cell cycle |
| MCM6 | Cell cycle |
| MCM7 | Cell cycle |
| MDM2 | Cell cycle |
| MYC | Cell cycle |
| ORC1 | Cell cycle |
| ORC2 | Cell cycle |
| ORC3 | Cell cycle |
| ORC4 | Cell cycle |
| ORC5 | Cell cycle |
| ORC6 | Cell cycle |
| PCNA | Cell cycle |
| PKMYT1 | Cell cycle |
| PLK1 | Cell cycle |
| PRKDC | Cell cycle |
| PTTG1 | Cell cycle |
| PTTG2 | Cell cycle |
| RAD21 | Cell cycle |
| RB1 | Cell cycle |
| RBL1 | Cell cycle |
| RBL2 | Cell cycle |
| RBX1 | Cell cycle |
| SFN | Cell cycle |
| SKP1 | Cell cycle |
| SKP2 | Cell cycle |
| SMAD2 | Cell cycle |
| SMAD3 | Cell cycle |
| SMAD4 | Cell cycle |
| SMC1A | Cell cycle |
| SMC1B | Cell cycle |
| SMC3 | Cell cycle |
| STAG1 | Cell cycle |
| STAG2 | Cell cycle |
| TFDP1 | Cell cycle |
| TFDP2 | Cell cycle |
| TGFB1 | Cell cycle |
| TGFB2 | Cell cycle |
| TGFB3 | Cell cycle |
| TP53 | Cell cycle |
| TTK | Cell cycle |
| WEE1 | Cell cycle |
| YWHAB | Cell cycle |
| YWHAE | Cell cycle |
| YWHAG | Cell cycle |
| YWHAH | Cell cycle |
| YWHAQ | Cell cycle |
| YWHAZ | Cell cycle |
| ZBTB17 | Cell cycle |
| STK4 | HIPPO |
| STK3 | HIPPO |
| SAV1 | HIPPO |
| LATS1 | HIPPO |
| LATS2 | HIPPO |
| MOB1A | HIPPO |
| MOB1B | HIPPO |
| YAP1 | HIPPO |
| WWTR1 | HIPPO |
| TEAD1 | HIPPO |
| TEAD2 | HIPPO |
| TEAD3 | HIPPO |
| TEAD4 | HIPPO |
| PTPN14 | HIPPO |
| NF2 | HIPPO |
| WWC1 | HIPPO |
| TAOK1 | HIPPO |
| TAOK2 | HIPPO |
| TAOK3 | HIPPO |
| CRB1 | HIPPO |
| CRB2 | HIPPO |
| CRB3 | HIPPO |
| LLGL1 | HIPPO |
| LLGL2 | HIPPO |
| HMCN1 | HIPPO |
| SCRIB | HIPPO |
| HIPK2 | HIPPO |
| FAT1 | HIPPO |
| FAT2 | HIPPO |
| FAT3 | HIPPO |
| FAT4 | HIPPO |
| DCHS1 | HIPPO |
| DCHS2 | HIPPO |
| CSNK1E | HIPPO |
| CSNK1D | HIPPO |
| AJUBA | HIPPO |
| LIMD1 | HIPPO |
| WTIP | HIPPO |
| MAX | MYC |
| MGA | MYC |
| MLX | MYC |
| MLXIP | MYC |
| MLXIPL | MYC |
| MNT | MYC |
| MXD1 | MYC |
| MXD3 | MYC |
| MXD4 | MYC |
| MXI1 | MYC |
| MYC | MYC |
| MYCL | MYC |
| MYCN | MYC |
| PCNA | MYC |
| PSMD8 | MYC |
| PSMD7 | MYC |
| SET | MYC |
| SNRPA1 | MYC |
| RAN | MYC |
| SRSF2 | MYC |
| G3BP1 | MYC |
| STARD7 | MYC |
| NPM1 | MYC |
| BUB3 | MYC |
| EIF3D | MYC |
| XPO1 | MYC |
| FBL | MYC |
| EIF4A1 | MYC |
| CANX | MYC |
| NAP1L1 | MYC |
| CBX3 | MYC |
| CCT3 | MYC |
| C1QBP | MYC |
| U2AF1 | MYC |
| UBE2L3 | MYC |
| SSBP1 | MYC |
| SRSF1 | MYC |
| TCP1 | MYC |
| MCM2 | MYC |
| EIF3B | MYC |
| PSMD14 | MYC |
| SNRPA | MYC |
| PWP1 | MYC |
| APEX1 | MYC |
| TXNL4A | MYC |
| HNRNPR | MYC |
| PSMB2 | MYC |
| HPRT1 | MYC |
| MCM6 | MYC |
| NME1 | MYC |
| SNRPD1 | MYC |
| EEF1B2 | MYC |
| HSPD1 | MYC |
| CAD | MYC |
| RPL18 | MYC |
| PGK1 | MYC |
| DDX18 | MYC |
| RPS2 | MYC |
| LDHA | MYC |
| RUVBL2 | MYC |
| RNPS1 | MYC |
| EIF2S1 | MYC |
| RANBP1 | MYC |
| MCM5 | MYC |
| IARS1 | MYC |
| UBE2E1 | MYC |
| AP3S1 | MYC |
| RFC4 | MYC |
| DUT | MYC |
| PSMA4 | MYC |
| RPS3 | MYC |
| SNRPG | MYC |
| PHB2 | MYC |
| SSB | MYC |
| EIF4H | MYC |
| SRSF3 | MYC |
| CCT4 | MYC |
| TFDP1 | MYC |
| EIF2S2 | MYC |
| CDK4 | MYC |
| SNRPD3 | MYC |
| PSMD1 | MYC |
| RACK1 | MYC |
| GOT2 | MYC |
| PABPC4 | MYC |
| CCT5 | MYC |
| PRPS2 | MYC |
| ACP1 | MYC |
| PRPF31 | MYC |
| CCT2 | MYC |
| KARS1 | MYC |
| DEK | MYC |
| EIF4G2 | MYC |
| XPOT | MYC |
| SRM | MYC |
| UBA2 | MYC |
| KPNB1 | MYC |
| PSMA7 | MYC |
| TRA2B | MYC |
| COX5A | MYC |
| PA2G4 | MYC |
| PCBP1 | MYC |
| HNRNPA1 | MYC |
| CCNA2 | MYC |
| PPIA | MYC |
| EIF3J | MYC |
| ORC2 | MYC |
| PSMA2 | MYC |
| SYNCRIP | MYC |
| HDAC2 | MYC |
| LSM2 | MYC |
| VBP1 | MYC |
| PSMA6 | MYC |
| CNBP | MYC |
| CDK2 | MYC |
| TARDBP | MYC |
| NOLC1 | MYC |
| GSPT1 | MYC |
| HNRNPA2B1 | MYC |
| ETF1 | MYC |
| RPL6 | MYC |
| TOMM70 | MYC |
| PTGES3 | MYC |
| PSMA1 | MYC |
| PSMC6 | MYC |
| CTPS1 | MYC |
| HSPE1 | MYC |
| FAM120A | MYC |
| PPM1G | MYC |
| HNRNPD | MYC |
| SERBP1 | MYC |
| CSTF2 | MYC |
| PRDX4 | MYC |
| EIF4E | MYC |
| ODC1 | MYC |
| USP1 | MYC |
| RPL22 | MYC |
| HNRNPA3 | MYC |
| NCBP1 | MYC |
| RPLP0 | MYC |
| ABCE1 | MYC |
| EIF1AX | MYC |
| YWHAE | MYC |
| MCM7 | MYC |
| YWHAQ | MYC |
| VDAC1 | MYC |
| SMARCC1 | MYC |
| SNRPD2 | MYC |
| HNRNPU | MYC |
| HDDC2 | MYC |
| PSMD3 | MYC |
| RRM1 | MYC |
| SF3A1 | MYC |
| LSM7 | MYC |
| ERH | MYC |
| RPL14 | MYC |
| PSMB3 | MYC |
| IFRD1 | MYC |
| NCBP2 | MYC |
| GLO1 | MYC |
| NDUFAB1 | MYC |
| CUL1 | MYC |
| NHP2 | MYC |
| CLNS1A | MYC |
| TRIM28 | MYC |
| RSL1D1 | MYC |
| HNRNPC | MYC |
| COPS5 | MYC |
| XRCC6 | MYC |
| SLC25A3 | MYC |
| MRPL9 | MYC |
| POLE3 | MYC |
| POLD2 | MYC |
| H2AZ1 | MYC |
| AIMP2 | MYC |
| NOP56 | MYC |
| PRDX3 | MYC |
| MRPS18B | MYC |
| EPRS1 | MYC |
| KPNA2 | MYC |
| HSP90AB1 | MYC |
| RPL34 | MYC |
| SRPK1 | MYC |
| MAD2L1 | MYC |
| DHX15 | MYC |
| MCM4 | MYC |
| RPS5 | MYC |
| CCT7 | MYC |
| HDGF | MYC |
| RPS6 | MYC |
| SNRPB2 | MYC |
| PSMC4 | MYC |
| CDC20 | MYC |
| TUFM | MYC |
| RRP9 | MYC |
| CDC45 | MYC |
| TYMS | MYC |
| ILF2 | MYC |
| VDAC3 | MYC |
| IMPDH2 | MYC |
| SF3B3 | MYC |
| NOP16 | MYC |
| SRSF7 | MYC |
| GNL3 | MYC |
| EXOSC7 | MYC |
| MRPL23 | MYC |
| RAD23B | MYC |
| RPS10 | MYC |
| PHB | MYC |
| DDX21 | MYC |
| CYC1 | MYC |
| PABPC1 | MYC |
| ARRDC1 | NOTCH |
| CNTN6 | NOTCH |
| CREBBP | NOTCH |
| EP300 | NOTCH |
| HES1 | NOTCH |
| HES2 | NOTCH |
| HES3 | NOTCH |
| HES4 | NOTCH |
| HES5 | NOTCH |
| HEY1 | NOTCH |
| HEY2 | NOTCH |
| HEYL | NOTCH |
| KAT2B | NOTCH |
| KDM5A | NOTCH |
| NOTCH1 | NOTCH |
| NOTCH2 | NOTCH |
| NOTCH3 | NOTCH |
| NOTCH4 | NOTCH |
| NOV | NOTCH |
| NRARP | NOTCH |
| PSEN2 | NOTCH |
| LFNG | NOTCH |
| ITCH | NOTCH |
| NCSTN | NOTCH |
| SPEN | NOTCH |
| JAG1 | NOTCH |
| APH1A | NOTCH |
| FBXW7 | NOTCH |
| FHL1 | NOTCH |
| THBS2 | NOTCH |
| HDAC2 | NOTCH |
| MFAP2 | NOTCH |
| CUL1 | NOTCH |
| RFNG | NOTCH |
| NCOR1 | NOTCH |
| NCOR2 | NOTCH |
| MFAP5 | NOTCH |
| HDAC1 | NOTCH |
| NUMB | NOTCH |
| JAG2 | NOTCH |
| MAML3 | NOTCH |
| MFNG | NOTCH |
| CIR1 | NOTCH |
| CNTN1 | NOTCH |
| MAML1 | NOTCH |
| MAML2 | NOTCH |
| NUMBL | NOTCH |
| PSEN1 | NOTCH |
| PSENEN | NOTCH |
| RBPJ | NOTCH |
| RBPJL | NOTCH |
| RBX1 | NOTCH |
| SAP30 | NOTCH |
| SKP1 | NOTCH |
| SNW1 | NOTCH |
| CTBP1 | NOTCH |
| CTBP2 | NOTCH |
| ADAM10 | NOTCH |
| APH1B | NOTCH |
| ADAM17 | NOTCH |
| DLK1 | NOTCH |
| DLL1 | NOTCH |
| DLL3 | NOTCH |
| DLL4 | NOTCH |
| DNER | NOTCH |
| DTX1 | NOTCH |
| DTX2 | NOTCH |
| DTX3 | NOTCH |
| DTX3L | NOTCH |
| DTX4 | NOTCH |
| EGFL7 | NOTCH |
| NFE2L2 | NRF2 |
| KEAP1 | NRF2 |
| CUL3 | NRF2 |
| EIF4EBP1 | PI3K |
| AKT1 | PI3K |
| AKT2 | PI3K |
| AKT3 | PI3K |
| AKT1S1 | PI3K |
| DEPDC5 | PI3K |
| DEPTOR | PI3K |
| INPP4B | PI3K |
| MAPKAP1 | PI3K |
| MLST8 | PI3K |
| MTOR | PI3K |
| NPRL2 | PI3K |
| NPRL3 | PI3K |
| PDK1 | PI3K |
| PIK3CA | PI3K |
| PIK3CB | PI3K |
| PIK3R1 | PI3K |
| PIK3R2 | PI3K |
| PIK3R3 | PI3K |
| PPP2R1A | PI3K |
| PTEN | PI3K |
| RHEB | PI3K |
| RICTOR | PI3K |
| RPTOR | PI3K |
| RPS6 | PI3K |
| RPS6KB1 | PI3K |
| STK11 | PI3K |
| TSC1 | PI3K |
| TSC2 | PI3K |
| GRB2 | PI3K |
| NFKBIB | PI3K |
| MAP2K6 | PI3K |
| MAPK9 | PI3K |
| MAPK1 | PI3K |
| PLCG1 | PI3K |
| TRIB3 | PI3K |
| GSK3B | PI3K |
| MAP2K3 | PI3K |
| CDKN1A | PI3K |
| RAC1 | PI3K |
| RIPK1 | PI3K |
| ACTR2 | PI3K |
| PRKAR2A | PI3K |
| YWHAB | PI3K |
| HRAS | PI3K |
| PIKFYVE | PI3K |
| TBK1 | PI3K |
| ACTR3 | PI3K |
| E2F1 | PI3K |
| MYD88 | PI3K |
| ITPR2 | PI3K |
| SQSTM1 | PI3K |
| RPS6KA1 | PI3K |
| PTPN11 | PI3K |
| PLCB1 | PI3K |
| RAF1 | PI3K |
| CAMK4 | PI3K |
| CFL1 | PI3K |
| CDK4 | PI3K |
| TRAF2 | PI3K |
| GNGT1 | PI3K |
| UBE2N | PI3K |
| ADCY2 | PI3K |
| CDKN1B | PI3K |
| VAV3 | PI3K |
| FGF6 | PI3K |
| ECSIT | PI3K |
| RALB | PI3K |
| ARF1 | PI3K |
| MKNK1 | PI3K |
| CDK1 | PI3K |
| ARHGDIA | PI3K |
| GRK2 | PI3K |
| FGF17 | PI3K |
| DDIT3 | PI3K |
| IRAK4 | PI3K |
| TIAM1 | PI3K |
| CDK2 | PI3K |
| SFN | PI3K |
| PRKCB | PI3K |
| GNA14 | PI3K |
| EIF4E | PI3K |
| CLTC | PI3K |
| FGF22 | PI3K |
| PPP1CA | PI3K |
| DUSP3 | PI3K |
| HSP90B1 | PI3K |
| IL4 | PI3K |
| STAT2 | PI3K |
| SLA | PI3K |
| EGFR | PI3K |
| PLA2G12A | PI3K |
| MAPK10 | PI3K |
| CALR | PI3K |
| THEM4 | PI3K |
| RIT1 | PI3K |
| MKNK2 | PI3K |
| PPP2R1B | PI3K |
| CAB39L | PI3K |
| ARPC3 | PI3K |
| PITX2 | PI3K |
| NCK1 | PI3K |
| IL2RG | PI3K |
| PFN1 | PI3K |
| FASLG | PI3K |
| NOD1 | PI3K |
| DAPP1 | PI3K |
| UBE2D3 | PI3K |
| CAB39 | PI3K |
| AP2M1 | PI3K |
| MAP3K7 | PI3K |
| PRKAG1 | PI3K |
| CSNK2B | PI3K |
| PRKAA2 | PI3K |
| ATF1 | PI3K |
| SLC2A1 | PI3K |
| PIN1 | PI3K |
| TNFRSF1A | PI3K |
| LCK | PI3K |
| RPS6KA3 | PI3K |
| NGF | PI3K |
| CXCR4 | PI3K |
| ACACA | PI3K |
| SMAD2 | PI3K |
| PAK4 | PI3K |
| TGFBR1 | TGF-Beta |
| TGFBR2 | TGF-Beta |
| ACVR2A | TGF-Beta |
| ACVR1B | TGF-Beta |
| SMAD2 | TGF-Beta |
| SMAD3 | TGF-Beta |
| SMAD4 | TGF-Beta |
| SPRED1 | RTK RAS |
| SPRED2 | RTK RAS |
| SPRED3 | RTK RAS |
| DAB2IP | RTK RAS |
| SHOC2 | RTK RAS |
| PPP1CA | RTK RAS |
| SCRIB | RTK RAS |
| PIN1 | RTK RAS |
| KSR1 | RTK RAS |
| KSR2 | RTK RAS |
| PEBP1 | RTK RAS |
| ERF | RTK RAS |
| PEA15 | RTK RAS |
| JAK2 | RTK RAS |
| IRS2 | RTK RAS |
| TP53 | TP53 pathway |
| MDM2 | TP53 pathway |
| MDM4 | TP53 pathway |
| ATM | TP53 pathway |
| CHEK2 | TP53 pathway |
| RPS6KA3 | TP53 pathway |
| CHD8 | WNT |
| LEF1 | WNT |
| LGR4 | WNT |
| LGR5 | WNT |
| LRP5 | WNT |
| LRP6 | WNT |
| LZTR1 | WNT |
| NDP | WNT |
| PORCN | WNT |
| RSPO1 | WNT |
| SFRP1 | WNT |
| SFRP2 | WNT |
| SFRP4 | WNT |
| SFRP5 | WNT |
| SOST | WNT |
| TCF7L1 | WNT |
| TLE1 | WNT |
| TLE2 | WNT |
| TLE3 | WNT |
| TLE4 | WNT |
| WIF1 | WNT |
| ZNRF3 | WNT |
| CTNNB1 | WNT |
| DVL1 | WNT |
| DVL2 | WNT |
| DVL3 | WNT |
| FRAT1 | WNT |
| FRAT2 | WNT |
| FZD1 | WNT |
| FZD10 | WNT |
| FZD2 | WNT |
| FZD3 | WNT |
| FZD4 | WNT |
| FZD5 | WNT |
| FZD6 | WNT |
| FZD7 | WNT |
| FZD8 | WNT |
| FZD9 | WNT |
| WNT1 | WNT |
| WNT10A | WNT |
| WNT10B | WNT |
| WNT11 | WNT |
| WNT16 | WNT |
| WNT2 | WNT |
| WNT3A | WNT |
| WNT4 | WNT |
| WNT5A | WNT |
| WNT5B | WNT |
| WNT6 | WNT |
| WNT7A | WNT |
| WNT7B | WNT |
| WNT8A | WNT |
| WNT8B | WNT |
| WNT9A | WNT |
| WNT9B | WNT |
| AMER1 | WNT |
| APC | WNT |
| AXIN1 | WNT |
| AXIN2 | WNT |
| DKK1 | WNT |
| DKK2 | WNT |
| DKK3 | WNT |
| DKK4 | WNT |
| GSK3B | WNT |
| RNF43 | WNT |
| TCF7 | WNT |
| TCF7L2 | WNT |
| CHD4 | WNT |
| EFNB3 | WNT |
| TCF12 | WNT |
| VEGFA | WNT |
| CLDN3 | EMT |
| CLDN7 | EMT |
| CLDN4 | EMT |
| CDH1 | EMT |
| VIM | EMT |
| TWIST1 | EMT |
| ZEB1 | EMT |
| ZEB2 | EMT |
| AXL | EMT |
| FAP | EMT |
| LOXL2 | EMT |
| ROR2 | EMT |
| TAGLN | EMT |
| TWIST2 | EMT |
| WNT5A | EMT |
| FOXF1 | EMT |
| GATA6 | EMT |
| SOX9 | EMT |
| TWIST1 | EMT |
| ZEB1 | EMT |
| ZEB2 | EMT |
| CDH5 | Angiogenesis |
| SOX17 | Angiogenesis |
| SOX18 | Angiogenesis |
| TEK | Angiogenesis |
| VEGFA | Angiogenesis |
| VEGFB | Angiogenesis |
| PGF | Angiogenesis |
| VEGFC | Angiogenesis |
| VEGFD | Angiogenesis |
| MMP2 | Angiogenesis |
| MMP1 | Angiogenesis |
| FGF2 | Angiogenesis |
| FGFR1 | Angiogenesis |
| FGFR2 | Angiogenesis |
| FGFR3 | Angiogenesis |
| FGFR4 | Angiogenesis |
| PDGFA | Angiogenesis |
| PDGFB | Angiogenesis |
| PDGFC | Angiogenesis |
| PDGFD | Angiogenesis |
| ANGPT1 | Angiogenesis |
| ANGPT2 | Angiogenesis |
| ANGPT4 | Angiogenesis |
| EPCAM | CSCs activity |
| PROM1 | CSCs activity |
| CD44 | CSCs activity |
| ANPEP | CSCs activity |
| THY1 | CSCs activity |
| KRT19 | CSCs activity |
| DLK1 | CSCs activity |
| IGDCC4 | CSCs activity |
| DCLK1 | CSCs activity |
| NANOG | CSCs activity |
| SOX9 | CSCs activity |
|  |  |

| **Table S2** The cell type gene sets used for immune cell infiltration analysis | | |
| --- | --- | --- |
| Metagene | Cell.type | Immunity |
| ADAM28 | Activated.B.cell | Adaptive |
| CD180 | Activated.B.cell | Adaptive |
| CD79B | Activated.B.cell | Adaptive |
| BLK | Activated.B.cell | Adaptive |
| CD19 | Activated.B.cell | Adaptive |
| MS4A1 | Activated.B.cell | Adaptive |
| TNFRSF17 | Activated.B.cell | Adaptive |
| IGHM | Activated.B.cell | Adaptive |
| GNG7 | Activated.B.cell | Adaptive |
| MICAL3 | Activated.B.cell | Adaptive |
| SPIB | Activated.B.cell | Adaptive |
| HLA-DOB | Activated.B.cell | Adaptive |
| IGKC | Activated.B.cell | Adaptive |
| PNOC | Activated.B.cell | Adaptive |
| FCRL2 | Activated.B.cell | Adaptive |
| BACH2 | Activated.B.cell | Adaptive |
| CR2 | Activated.B.cell | Adaptive |
| TCL1A | Activated.B.cell | Adaptive |
| AKNA | Activated.B.cell | Adaptive |
| ARHGAP25 | Activated.B.cell | Adaptive |
| CCL21 | Activated.B.cell | Adaptive |
| CD27 | Activated.B.cell | Adaptive |
| CD38 | Activated.B.cell | Adaptive |
| CLEC17A | Activated.B.cell | Adaptive |
| CLEC9A | Activated.B.cell | Adaptive |
| CLECL1 | Activated.B.cell | Adaptive |
| AIM2 | Activated.CD4.T.cell | Adaptive |
| BIRC3 | Activated.CD4.T.cell | Adaptive |
| BRIP1 | Activated.CD4.T.cell | Adaptive |
| CCL20 | Activated.CD4.T.cell | Adaptive |
| CCL4 | Activated.CD4.T.cell | Adaptive |
| CCL5 | Activated.CD4.T.cell | Adaptive |
| CCNB1 | Activated.CD4.T.cell | Adaptive |
| CCR7 | Activated.CD4.T.cell | Adaptive |
| DUSP2 | Activated.CD4.T.cell | Adaptive |
| ESCO2 | Activated.CD4.T.cell | Adaptive |
| ETS1 | Activated.CD4.T.cell | Adaptive |
| EXO1 | Activated.CD4.T.cell | Adaptive |
| EXOC6 | Activated.CD4.T.cell | Adaptive |
| IARS | Activated.CD4.T.cell | Adaptive |
| ITK | Activated.CD4.T.cell | Adaptive |
| KIF11 | Activated.CD4.T.cell | Adaptive |
| KNTC1 | Activated.CD4.T.cell | Adaptive |
| NUF2 | Activated.CD4.T.cell | Adaptive |
| PRC1 | Activated.CD4.T.cell | Adaptive |
| PSAT1 | Activated.CD4.T.cell | Adaptive |
| RGS1 | Activated.CD4.T.cell | Adaptive |
| RTKN2 | Activated.CD4.T.cell | Adaptive |
| SAMSN1 | Activated.CD4.T.cell | Adaptive |
| SELL | Activated.CD4.T.cell | Adaptive |
| TRAT1 | Activated.CD4.T.cell | Adaptive |
| ADRM1 | Activated.CD8.T.cell | Adaptive |
| AHSA1 | Activated.CD8.T.cell | Adaptive |
| C1GALT1C1 | Activated.CD8.T.cell | Adaptive |
| CCT6B | Activated.CD8.T.cell | Adaptive |
| CD37 | Activated.CD8.T.cell | Adaptive |
| CD3D | Activated.CD8.T.cell | Adaptive |
| CD3E | Activated.CD8.T.cell | Adaptive |
| CD3G | Activated.CD8.T.cell | Adaptive |
| CD69 | Activated.CD8.T.cell | Adaptive |
| CD8A | Activated.CD8.T.cell | Adaptive |
| CETN3 | Activated.CD8.T.cell | Adaptive |
| CSE1L | Activated.CD8.T.cell | Adaptive |
| GEMIN6 | Activated.CD8.T.cell | Adaptive |
| GNLY | Activated.CD8.T.cell | Adaptive |
| GPT2 | Activated.CD8.T.cell | Adaptive |
| GZMA | Activated.CD8.T.cell | Adaptive |
| GZMH | Activated.CD8.T.cell | Adaptive |
| GZMK | Activated.CD8.T.cell | Adaptive |
| IL2RB | Activated.CD8.T.cell | Adaptive |
| LCK | Activated.CD8.T.cell | Adaptive |
| MPZL1 | Activated.CD8.T.cell | Adaptive |
| NKG7 | Activated.CD8.T.cell | Adaptive |
| PIK3IP1 | Activated.CD8.T.cell | Adaptive |
| PTRH2 | Activated.CD8.T.cell | Adaptive |
| TIMM13 | Activated.CD8.T.cell | Adaptive |
| ZAP70 | Activated.CD8.T.cell | Adaptive |
| ACP5 | Gamma.delta.T.cell | Adaptive |
| AQP9 | Gamma.delta.T.cell | Adaptive |
| BTN3A2 | Gamma.delta.T.cell | Adaptive |
| C1orf54 | Gamma.delta.T.cell | Adaptive |
| CARD8 | Gamma.delta.T.cell | Adaptive |
| CCL18 | Gamma.delta.T.cell | Adaptive |
| CD209 | Gamma.delta.T.cell | Adaptive |
| CD33 | Gamma.delta.T.cell | Adaptive |
| CD36 | Gamma.delta.T.cell | Adaptive |
| CDK5 | Gamma.delta.T.cell | Adaptive |
| IL10RB | Gamma.delta.T.cell | Adaptive |
| KLRF1 | Gamma.delta.T.cell | Adaptive |
| LGALS1 | Gamma.delta.T.cell | Adaptive |
| MAPK7 | Gamma.delta.T.cell | Adaptive |
| KLHL7 | Gamma.delta.T.cell | Adaptive |
| KRT80 | Gamma.delta.T.cell | Adaptive |
| LAMC1 | Gamma.delta.T.cell | Adaptive |
| LCORL | Gamma.delta.T.cell | Adaptive |
| LMNB1 | Gamma.delta.T.cell | Adaptive |
| MEIS3P1 | Gamma.delta.T.cell | Adaptive |
| MPL | Gamma.delta.T.cell | Adaptive |
| FABP1 | Gamma.delta.T.cell | Adaptive |
| FABP5 | Gamma.delta.T.cell | Adaptive |
| FADD | Gamma.delta.T.cell | Adaptive |
| MFAP3L | Gamma.delta.T.cell | Adaptive |
| MINPP1 | Gamma.delta.T.cell | Adaptive |
| RPS24 | Gamma.delta.T.cell | Adaptive |
| RPS7 | Gamma.delta.T.cell | Adaptive |
| RPS9 | Gamma.delta.T.cell | Adaptive |
| DBNL | Gamma.delta.T.cell | Adaptive |
| CCL13 | Gamma.delta.T.cell | Adaptive |
| CD22 | Immature..B.cell | Adaptive |
| CYBB | Immature..B.cell | Adaptive |
| FAM129C | Immature..B.cell | Adaptive |
| FCRL1 | Immature..B.cell | Adaptive |
| FCRL3 | Immature..B.cell | Adaptive |
| FCRL5 | Immature..B.cell | Adaptive |
| FCRLA | Immature..B.cell | Adaptive |
| HDAC9 | Immature..B.cell | Adaptive |
| HLA-DQA1 | Immature..B.cell | Adaptive |
| HVCN1 | Immature..B.cell | Adaptive |
| KIAA0226 | Immature..B.cell | Adaptive |
| NCF1 | Immature..B.cell | Adaptive |
| NCF1B | Immature..B.cell | Adaptive |
| P2RY10 | Immature..B.cell | Adaptive |
| SP100 | Immature..B.cell | Adaptive |
| TXNIP | Immature..B.cell | Adaptive |
| STAP1 | Immature..B.cell | Adaptive |
| TAGAP | Immature..B.cell | Adaptive |
| ZCCHC2 | Immature..B.cell | Adaptive |
| CCL3L1 | Regulatory.T.cell | Adaptive |
| CD72 | Regulatory.T.cell | Adaptive |
| CLEC5A | Regulatory.T.cell | Adaptive |
| FOXP3 | Regulatory.T.cell | Adaptive |
| ITGA4 | Regulatory.T.cell | Adaptive |
| L1CAM | Regulatory.T.cell | Adaptive |
| LIPA | Regulatory.T.cell | Adaptive |
| LRP1 | Regulatory.T.cell | Adaptive |
| LRRC42 | Regulatory.T.cell | Adaptive |
| MARCO | Regulatory.T.cell | Adaptive |
| MMP12 | Regulatory.T.cell | Adaptive |
| MNDA | Regulatory.T.cell | Adaptive |
| MRC1 | Regulatory.T.cell | Adaptive |
| MS4A6A | Regulatory.T.cell | Adaptive |
| PELO | Regulatory.T.cell | Adaptive |
| PLEK | Regulatory.T.cell | Adaptive |
| PRSS23 | Regulatory.T.cell | Adaptive |
| PTGIR | Regulatory.T.cell | Adaptive |
| ST8SIA4 | Regulatory.T.cell | Adaptive |
| STAB1 | Regulatory.T.cell | Adaptive |
| B3GAT1 | T.follicular.helper.cell | Adaptive |
| CDK5R1 | T.follicular.helper.cell | Adaptive |
| PDCD1 | T.follicular.helper.cell | Adaptive |
| BCL6 | T.follicular.helper.cell | Adaptive |
| CD200 | T.follicular.helper.cell | Adaptive |
| CD83 | T.follicular.helper.cell | Adaptive |
| CD84 | T.follicular.helper.cell | Adaptive |
| FGF2 | T.follicular.helper.cell | Adaptive |
| GPR18 | T.follicular.helper.cell | Adaptive |
| CEBPA | T.follicular.helper.cell | Adaptive |
| CECR1 | T.follicular.helper.cell | Adaptive |
| CLEC10A | T.follicular.helper.cell | Adaptive |
| CLEC4A | T.follicular.helper.cell | Adaptive |
| CSF1R | T.follicular.helper.cell | Adaptive |
| CTSS | T.follicular.helper.cell | Adaptive |
| DMN | T.follicular.helper.cell | Adaptive |
| DPP4 | T.follicular.helper.cell | Adaptive |
| LRRC32 | T.follicular.helper.cell | Adaptive |
| MC5R | T.follicular.helper.cell | Adaptive |
| MICA | T.follicular.helper.cell | Adaptive |
| NCAM1 | T.follicular.helper.cell | Adaptive |
| NCR2 | T.follicular.helper.cell | Adaptive |
| NRP1 | T.follicular.helper.cell | Adaptive |
| PDCD1LG2 | T.follicular.helper.cell | Adaptive |
| PDCD6 | T.follicular.helper.cell | Adaptive |
| PRDX1 | T.follicular.helper.cell | Adaptive |
| RAE1 | T.follicular.helper.cell | Adaptive |
| RAET1E | T.follicular.helper.cell | Adaptive |
| SIGLEC7 | T.follicular.helper.cell | Adaptive |
| SIGLEC9 | T.follicular.helper.cell | Adaptive |
| TYRO3 | T.follicular.helper.cell | Adaptive |
| CHST12 | T.follicular.helper.cell | Adaptive |
| CLIC3 | T.follicular.helper.cell | Adaptive |
| IVNS1ABP | T.follicular.helper.cell | Adaptive |
| KIR2DL2 | T.follicular.helper.cell | Adaptive |
| LGMN | T.follicular.helper.cell | Adaptive |
| CD70 | Type.1.T.helper.cell | Adaptive |
| TBX21 | Type.1.T.helper.cell | Adaptive |
| ADAM8 | Type.1.T.helper.cell | Adaptive |
| AHCYL2 | Type.1.T.helper.cell | Adaptive |
| ALCAM | Type.1.T.helper.cell | Adaptive |
| B3GALNT1 | Type.1.T.helper.cell | Adaptive |
| BBS12 | Type.1.T.helper.cell | Adaptive |
| BST1 | Type.1.T.helper.cell | Adaptive |
| CD151 | Type.1.T.helper.cell | Adaptive |
| CD47 | Type.1.T.helper.cell | Adaptive |
| CD48 | Type.1.T.helper.cell | Adaptive |
| CD52 | Type.1.T.helper.cell | Adaptive |
| CD53 | Type.1.T.helper.cell | Adaptive |
| CD59 | Type.1.T.helper.cell | Adaptive |
| CD6 | Type.1.T.helper.cell | Adaptive |
| CD68 | Type.1.T.helper.cell | Adaptive |
| CD7 | Type.1.T.helper.cell | Adaptive |
| CD96 | Type.1.T.helper.cell | Adaptive |
| CFHR3 | Type.1.T.helper.cell | Adaptive |
| CHRM3 | Type.1.T.helper.cell | Adaptive |
| CLEC7A | Type.1.T.helper.cell | Adaptive |
| COL23A1 | Type.1.T.helper.cell | Adaptive |
| COL4A4 | Type.1.T.helper.cell | Adaptive |
| COL5A3 | Type.1.T.helper.cell | Adaptive |
| DAB1 | Type.1.T.helper.cell | Adaptive |
| DLEU7 | Type.1.T.helper.cell | Adaptive |
| DOC2B | Type.1.T.helper.cell | Adaptive |
| EMP1 | Type.1.T.helper.cell | Adaptive |
| F12 | Type.1.T.helper.cell | Adaptive |
| FURIN | Type.1.T.helper.cell | Adaptive |
| GAB3 | Type.1.T.helper.cell | Adaptive |
| GATM | Type.1.T.helper.cell | Adaptive |
| GFPT2 | Type.1.T.helper.cell | Adaptive |
| GPR25 | Type.1.T.helper.cell | Adaptive |
| GREM2 | Type.1.T.helper.cell | Adaptive |
| HAVCR1 | Type.1.T.helper.cell | Adaptive |
| HSD11B1 | Type.1.T.helper.cell | Adaptive |
| HUNK | Type.1.T.helper.cell | Adaptive |
| IGF2 | Type.1.T.helper.cell | Adaptive |
| RCSD1 | Type.1.T.helper.cell | Adaptive |
| RYR1 | Type.1.T.helper.cell | Adaptive |
| SAV1 | Type.1.T.helper.cell | Adaptive |
| SELE | Type.1.T.helper.cell | Adaptive |
| SELP | Type.1.T.helper.cell | Adaptive |
| SH3KBP1 | Type.1.T.helper.cell | Adaptive |
| SIT1 | Type.1.T.helper.cell | Adaptive |
| SLC35B3 | Type.1.T.helper.cell | Adaptive |
| SIGLEC10 | Type.1.T.helper.cell | Adaptive |
| SKAP1 | Type.1.T.helper.cell | Adaptive |
| THUMPD2 | Type.1.T.helper.cell | Adaptive |
| TIGIT | Type.1.T.helper.cell | Adaptive |
| ZEB2 | Type.1.T.helper.cell | Adaptive |
| ENC1 | Type.1.T.helper.cell | Adaptive |
| FAM134B | Type.1.T.helper.cell | Adaptive |
| FBXO30 | Type.1.T.helper.cell | Adaptive |
| FCGR2C | Type.1.T.helper.cell | Adaptive |
| STAC | Type.1.T.helper.cell | Adaptive |
| LTC4S | Type.1.T.helper.cell | Adaptive |
| MAN1B1 | Type.1.T.helper.cell | Adaptive |
| MDH1 | Type.1.T.helper.cell | Adaptive |
| MMD | Type.1.T.helper.cell | Adaptive |
| RGS16 | Type.1.T.helper.cell | Adaptive |
| IL12A | Type.1.T.helper.cell | Adaptive |
| P2RX5 | Type.1.T.helper.cell | Adaptive |
| CD97 | Type.1.T.helper.cell | Adaptive |
| ITGB4 | Type.1.T.helper.cell | Adaptive |
| ICAM3 | Type.1.T.helper.cell | Adaptive |
| METRNL | Type.1.T.helper.cell | Adaptive |
| TNFRSF1A | Type.1.T.helper.cell | Adaptive |
| IRF1 | Type.1.T.helper.cell | Adaptive |
| HTR2B | Type.1.T.helper.cell | Adaptive |
| CALD1 | Type.1.T.helper.cell | Adaptive |
| MOCOS | Type.1.T.helper.cell | Adaptive |
| TRAF3IP2 | Type.1.T.helper.cell | Adaptive |
| TLR8 | Type.1.T.helper.cell | Adaptive |
| TRAF1 | Type.1.T.helper.cell | Adaptive |
| DUSP14 | Type.1.T.helper.cell | Adaptive |
| IL17A | Type.17.T.helper.cell | Adaptive |
| IL17RA | Type.17.T.helper.cell | Adaptive |
| C2CD4A | Type.17.T.helper.cell | Adaptive |
| C2CD4B | Type.17.T.helper.cell | Adaptive |
| CA2 | Type.17.T.helper.cell | Adaptive |
| CCDC65 | Type.17.T.helper.cell | Adaptive |
| CEACAM3 | Type.17.T.helper.cell | Adaptive |
| IL17C | Type.17.T.helper.cell | Adaptive |
| IL17F | Type.17.T.helper.cell | Adaptive |
| IL17RC | Type.17.T.helper.cell | Adaptive |
| IL17RE | Type.17.T.helper.cell | Adaptive |
| IL23A | Type.17.T.helper.cell | Adaptive |
| ILDR1 | Type.17.T.helper.cell | Adaptive |
| LONRF3 | Type.17.T.helper.cell | Adaptive |
| SH2D6 | Type.17.T.helper.cell | Adaptive |
| TNIP2 | Type.17.T.helper.cell | Adaptive |
| ABCA1 | Type.17.T.helper.cell | Adaptive |
| ABCB1 | Type.17.T.helper.cell | Adaptive |
| ADAMTS12 | Type.17.T.helper.cell | Adaptive |
| ANK1 | Type.17.T.helper.cell | Adaptive |
| ANKRD22 | Type.17.T.helper.cell | Adaptive |
| B3GALT2 | Type.17.T.helper.cell | Adaptive |
| CAMTA1 | Type.17.T.helper.cell | Adaptive |
| CCR9 | Type.17.T.helper.cell | Adaptive |
| CD40 | Type.17.T.helper.cell | Adaptive |
| GPR44 | Type.17.T.helper.cell | Adaptive |
| IFT80 | Type.17.T.helper.cell | Adaptive |
| ASB2 | Type.2.T.helper.cell | Adaptive |
| CSRP2 | Type.2.T.helper.cell | Adaptive |
| DAPK1 | Type.2.T.helper.cell | Adaptive |
| DLC1 | Type.2.T.helper.cell | Adaptive |
| DNAJC12 | Type.2.T.helper.cell | Adaptive |
| DUSP6 | Type.2.T.helper.cell | Adaptive |
| GNAI1 | Type.2.T.helper.cell | Adaptive |
| LAMP3 | Type.2.T.helper.cell | Adaptive |
| NRP2 | Type.2.T.helper.cell | Adaptive |
| OSBPL1A | Type.2.T.helper.cell | Adaptive |
| PDE4B | Type.2.T.helper.cell | Adaptive |
| PHLDA1 | Type.2.T.helper.cell | Adaptive |
| PLA2G4A | Type.2.T.helper.cell | Adaptive |
| RAB27B | Type.2.T.helper.cell | Adaptive |
| RBMS3 | Type.2.T.helper.cell | Adaptive |
| RNF125 | Type.2.T.helper.cell | Adaptive |
| TMPRSS3 | Type.2.T.helper.cell | Adaptive |
| GATA3 | Type.2.T.helper.cell | Adaptive |
| BIRC5 | Type.2.T.helper.cell | Adaptive |
| CDC25C | Type.2.T.helper.cell | Adaptive |
| CDC7 | Type.2.T.helper.cell | Adaptive |
| CENPF | Type.2.T.helper.cell | Adaptive |
| CXCR6 | Type.2.T.helper.cell | Adaptive |
| DHFR | Type.2.T.helper.cell | Adaptive |
| EVI5 | Type.2.T.helper.cell | Adaptive |
| GSTA4 | Type.2.T.helper.cell | Adaptive |
| HELLS | Type.2.T.helper.cell | Adaptive |
| IL26 | Type.2.T.helper.cell | Adaptive |
| LAIR2 | Type.2.T.helper.cell | Adaptive |
| ABCD1 | Activated.dendritic.cell | Innate |
| C1QC | Activated.dendritic.cell | Innate |
| CAPG | Activated.dendritic.cell | Innate |
| CCL3L3 | Activated.dendritic.cell | Innate |
| CD207 | Activated.dendritic.cell | Innate |
| CD302 | Activated.dendritic.cell | Innate |
| ATP5B | Activated.dendritic.cell | Innate |
| ATP5L | Activated.dendritic.cell | Innate |
| ATP6V1A | Activated.dendritic.cell | Innate |
| BCL2L1 | Activated.dendritic.cell | Innate |
| C1QB | Activated.dendritic.cell | Innate |
| SNURF | Activated.dendritic.cell | Innate |
| SPCS3 | Activated.dendritic.cell | Innate |
| CCNA1 | Activated.dendritic.cell | Innate |
| CEACAM8 | Activated.dendritic.cell | Innate |
| NOS2 | Activated.dendritic.cell | Innate |
| SRA1 | Activated.dendritic.cell | Innate |
| TNFRSF6B | Activated.dendritic.cell | Innate |
| TREM1 | Activated.dendritic.cell | Innate |
| TREML1 | Activated.dendritic.cell | Innate |
| RHOA | Activated.dendritic.cell | Innate |
| SLC25A37 | Activated.dendritic.cell | Innate |
| TNFSF14 | Activated.dendritic.cell | Innate |
| TREML4 | Activated.dendritic.cell | Innate |
| VNN2 | Activated.dendritic.cell | Innate |
| XPO6 | Activated.dendritic.cell | Innate |
| CLEC4C | Activated.dendritic.cell | Innate |
| TNFAIP2 | Activated.dendritic.cell | Innate |
| UBD | Activated.dendritic.cell | Innate |
| ACTR3 | Activated.dendritic.cell | Innate |
| RAB1A | Activated.dendritic.cell | Innate |
| SLA | Activated.dendritic.cell | Innate |
| HLA-DQA2 | Activated.dendritic.cell | Innate |
| SIGLEC5 | Activated.dendritic.cell | Innate |
| SLAMF9 | Activated.dendritic.cell | Innate |
| ABAT | CD56bright.natural.killer.cell | Innate |
| C11orf75 | CD56bright.natural.killer.cell | Innate |
| C5orf15 | CD56bright.natural.killer.cell | Innate |
| CDHR1 | CD56bright.natural.killer.cell | Innate |
| DCAF12 | CD56bright.natural.killer.cell | Innate |
| DYNLL1 | CD56bright.natural.killer.cell | Innate |
| GPR137B | CD56bright.natural.killer.cell | Innate |
| HCP5 | CD56bright.natural.killer.cell | Innate |
| HDGFRP2 | CD56bright.natural.killer.cell | Innate |
| KRT86 | CD56bright.natural.killer.cell | Innate |
| MLST8 | CD56bright.natural.killer.cell | Innate |
| ELMOD3 | CD56bright.natural.killer.cell | Innate |
| ENTPD5 | CD56bright.natural.killer.cell | Innate |
| FAM119A | CD56bright.natural.killer.cell | Innate |
| FAM179A | CD56bright.natural.killer.cell | Innate |
| CLIC2 | CD56bright.natural.killer.cell | Innate |
| COX7A2L | CD56bright.natural.killer.cell | Innate |
| CREB3L4 | CD56bright.natural.killer.cell | Innate |
| CSF1 | CD56bright.natural.killer.cell | Innate |
| CSNK2A2 | CD56bright.natural.killer.cell | Innate |
| CSTA | CD56bright.natural.killer.cell | Innate |
| CSTB | CD56bright.natural.killer.cell | Innate |
| CTPS | CD56bright.natural.killer.cell | Innate |
| CTSD | CD56bright.natural.killer.cell | Innate |
| FST | CD56bright.natural.killer.cell | Innate |
| GATA2 | CD56bright.natural.killer.cell | Innate |
| GMPR | CD56bright.natural.killer.cell | Innate |
| HDC | CD56bright.natural.killer.cell | Innate |
| HEY1 | CD56bright.natural.killer.cell | Innate |
| HOXA1 | CD56bright.natural.killer.cell | Innate |
| HS2ST1 | CD56bright.natural.killer.cell | Innate |
| HS3ST1 | CD56bright.natural.killer.cell | Innate |
| BCL11B | CD56bright.natural.killer.cell | Innate |
| CDH3 | CD56bright.natural.killer.cell | Innate |
| MYL6B | CD56bright.natural.killer.cell | Innate |
| NAA16 | CD56bright.natural.killer.cell | Innate |
| ClQA | CD56bright.natural.killer.cell | Innate |
| ClQB | CD56bright.natural.killer.cell | Innate |
| CYP27B1 | CD56bright.natural.killer.cell | Innate |
| EIF3M | CD56bright.natural.killer.cell | Innate |
| CYP27A1 | CD56dim.natural.killer.cell | Innate |
| DDX55 | CD56dim.natural.killer.cell | Innate |
| DYRK2 | CD56dim.natural.killer.cell | Innate |
| RPL37A | CD56dim.natural.killer.cell | Innate |
| NOTCH3 | CD56dim.natural.killer.cell | Innate |
| AKR7A3 | CD56dim.natural.killer.cell | Innate |
| GPRC5C | CD56dim.natural.killer.cell | Innate |
| GRIN1 | CD56dim.natural.killer.cell | Innate |
| HLA-E | CD56dim.natural.killer.cell | Innate |
| PORCN | CD56dim.natural.killer.cell | Innate |
| PSMC4 | CD56dim.natural.killer.cell | Innate |
| UPP1 | CD56dim.natural.killer.cell | Innate |
| IL21R | CD56dim.natural.killer.cell | Innate |
| KIR2DS1 | CD56dim.natural.killer.cell | Innate |
| KIR2DS2 | CD56dim.natural.killer.cell | Innate |
| KIR2DS5 | CD56dim.natural.killer.cell | Innate |
| GIPR | Eosinophil | Innate |
| KRT18P50 | Eosinophil | Innate |
| LRMP | Eosinophil | Innate |
| FOSB | Eosinophil | Innate |
| RRP12 | Eosinophil | Innate |
| GPR183 | Eosinophil | Innate |
| NR4A3 | Eosinophil | Innate |
| ST3GAL6 | Eosinophil | Innate |
| DEPDC5 | Eosinophil | Innate |
| PDE6C | Eosinophil | Innate |
| PKD2L2 | Eosinophil | Innate |
| GPR65 | Eosinophil | Innate |
| IL5RA | Eosinophil | Innate |
| P2RY14 | Eosinophil | Innate |
| DACH1 | Eosinophil | Innate |
| DAPK2 | Eosinophil | Innate |
| EMR3 | Eosinophil | Innate |
| ACADM | Immature.dendritic.cell | Innate |
| AHCYL1 | Immature.dendritic.cell | Innate |
| ALDH1A2 | Immature.dendritic.cell | Innate |
| ALDH3A2 | Immature.dendritic.cell | Innate |
| ALDH9A1 | Immature.dendritic.cell | Innate |
| ALOX15 | Immature.dendritic.cell | Innate |
| AMT | Immature.dendritic.cell | Innate |
| ARL1 | Immature.dendritic.cell | Innate |
| ATIC | Immature.dendritic.cell | Innate |
| ATP5A1 | Immature.dendritic.cell | Innate |
| CAPZA1 | Immature.dendritic.cell | Innate |
| LILRA5 | Immature.dendritic.cell | Innate |
| RDX | Immature.dendritic.cell | Innate |
| RRAGD | Immature.dendritic.cell | Innate |
| TACSTD2 | Immature.dendritic.cell | Innate |
| INPP5F | Immature.dendritic.cell | Innate |
| RAB38 | Immature.dendritic.cell | Innate |
| PLAU | Immature.dendritic.cell | Innate |
| CSF3R | Immature.dendritic.cell | Innate |
| SLC18A2 | Immature.dendritic.cell | Innate |
| AMPD2 | Immature.dendritic.cell | Innate |
| CLTB | Immature.dendritic.cell | Innate |
| C1orf162 | Immature.dendritic.cell | Innate |
| AIF1 | Macrophage | Innate |
| CCL1 | Macrophage | Innate |
| CCL14 | Macrophage | Innate |
| CCL23 | Macrophage | Innate |
| CCL26 | Macrophage | Innate |
| CD300LB | Macrophage | Innate |
| CNR1 | Macrophage | Innate |
| CNR2 | Macrophage | Innate |
| EIF1 | Macrophage | Innate |
| EIF4A1 | Macrophage | Innate |
| FPR1 | Macrophage | Innate |
| FPR2 | Macrophage | Innate |
| FRAT2 | Macrophage | Innate |
| GPR27 | Macrophage | Innate |
| GPR77 | Macrophage | Innate |
| RNASE2 | Macrophage | Innate |
| MS4A2 | Macrophage | Innate |
| BASP1 | Macrophage | Innate |
| IGSF6 | Macrophage | Innate |
| HK3 | Macrophage | Innate |
| VNN1 | Macrophage | Innate |
| FES | Macrophage | Innate |
| NPL | Macrophage | Innate |
| FZD2 | Macrophage | Innate |
| FAM198B | Macrophage | Innate |
| HNMT | Macrophage | Innate |
| SLC15A3 | Macrophage | Innate |
| CD4 | Macrophage | Innate |
| TXNDC3 | Macrophage | Innate |
| FRMD4A | Macrophage | Innate |
| CRYBB1 | Macrophage | Innate |
| HRH1 | Macrophage | Innate |
| WNT5B | Macrophage | Innate |
| ADAMTS3 | Mast.cell | Innate |
| CPA3 | Mast.cell | Innate |
| CMA1 | Mast.cell | Innate |
| CTSG | Mast.cell | Innate |
| ARHGAP15 | Mast.cell | Innate |
| CPM | Mast.cell | Innate |
| FCN1 | Mast.cell | Innate |
| FTL | Mast.cell | Innate |
| HSPA6 | Mast.cell | Innate |
| ITGA9 | Mast.cell | Innate |
| RNASE3 | Mast.cell | Innate |
| S100A4 | Mast.cell | Innate |
| SIGLEC8 | Mast.cell | Innate |
| SLC6A4 | Mast.cell | Innate |
| PTGS2 | Mast.cell | Innate |
| EGR3 | Mast.cell | Innate |
| PILRA | Mast.cell | Innate |
| CCR2 | MDSC | Innate |
| CD14 | MDSC | Innate |
| CD2 | MDSC | Innate |
| CD86 | MDSC | Innate |
| CXCR4 | MDSC | Innate |
| FCGR2A | MDSC | Innate |
| FCGR2B | MDSC | Innate |
| FCGR3A | MDSC | Innate |
| FERMT3 | MDSC | Innate |
| GPSM3 | MDSC | Innate |
| IL18BP | MDSC | Innate |
| IL4R | MDSC | Innate |
| ITGAL | MDSC | Innate |
| ITGAM | MDSC | Innate |
| PARVG | MDSC | Innate |
| PSAP | MDSC | Innate |
| PTGER2 | MDSC | Innate |
| PTGES2 | MDSC | Innate |
| S100A8 | MDSC | Innate |
| S100A9 | MDSC | Innate |
| ASGR2 | Monocyte | Innate |
| CFP | Monocyte | Innate |
| ASGR1 | Monocyte | Innate |
| CD1D | Monocyte | Innate |
| UPK3A | Monocyte | Innate |
| ACTG1 | Monocyte | Innate |
| ANXA5 | Monocyte | Innate |
| ATP6V1B2 | Monocyte | Innate |
| CFL1 | Monocyte | Innate |
| DAZAP2 | Monocyte | Innate |
| CTBS | Monocyte | Innate |
| EMR4P | Monocyte | Innate |
| HIVEP2 | Monocyte | Innate |
| MARCKSL1 | Monocyte | Innate |
| MBP | Monocyte | Innate |
| MMP15 | Monocyte | Innate |
| PNPLA6 | Monocyte | Innate |
| TMBIM6 | Monocyte | Innate |
| PQBP1 | Monocyte | Innate |
| TEX264 | Monocyte | Innate |
| IKZF1 | Monocyte | Innate |
| AKT3 | Natural.killer.cell | Innate |
| AXL | Natural.killer.cell | Innate |
| BST2 | Natural.killer.cell | Innate |
| CDH2 | Natural.killer.cell | Innate |
| CRTAM | Natural.killer.cell | Innate |
| CSF2RA | Natural.killer.cell | Innate |
| CTSZ | Natural.killer.cell | Innate |
| CXCL1 | Natural.killer.cell | Innate |
| CYTH1 | Natural.killer.cell | Innate |
| DAXX | Natural.killer.cell | Innate |
| DGKH | Natural.killer.cell | Innate |
| DLL4 | Natural.killer.cell | Innate |
| DPYD | Natural.killer.cell | Innate |
| ERBB3 | Natural.killer.cell | Innate |
| F11R | Natural.killer.cell | Innate |
| FAM27A | Natural.killer.cell | Innate |
| FAM49A | Natural.killer.cell | Innate |
| FASLG | Natural.killer.cell | Innate |
| FCGR1A | Natural.killer.cell | Innate |
| FN1 | Natural.killer.cell | Innate |
| FSTL1 | Natural.killer.cell | Innate |
| FUCA1 | Natural.killer.cell | Innate |
| GBP3 | Natural.killer.cell | Innate |
| GLS2 | Natural.killer.cell | Innate |
| GRB2 | Natural.killer.cell | Innate |
| LST1 | Natural.killer.cell | Innate |
| BCL2 | Natural.killer.cell | Innate |
| CDC5L | Natural.killer.cell | Innate |
| FGF18 | Natural.killer.cell | Innate |
| FUT5 | Natural.killer.cell | Innate |
| FZR1 | Natural.killer.cell | Innate |
| GAGE2 | Natural.killer.cell | Innate |
| IGFBP5 | Natural.killer.cell | Innate |
| KANK2 | Natural.killer.cell | Innate |
| LDB3 | Natural.killer.cell | Innate |
| BTN2A2 | Natural.killer.T.cell | Innate |
| CD101 | Natural.killer.T.cell | Innate |
| CD109 | Natural.killer.T.cell | Innate |
| CNPY3 | Natural.killer.T.cell | Innate |
| CNPY4 | Natural.killer.T.cell | Innate |
| CREB1 | Natural.killer.T.cell | Innate |
| CRTC2 | Natural.killer.T.cell | Innate |
| CRTC3 | Natural.killer.T.cell | Innate |
| CSF2 | Natural.killer.T.cell | Innate |
| KLRC1 | Natural.killer.T.cell | Innate |
| FUT4 | Natural.killer.T.cell | Innate |
| ICAM2 | Natural.killer.T.cell | Innate |
| IL32 | Natural.killer.T.cell | Innate |
| LAMP2 | Natural.killer.T.cell | Innate |
| LILRB5 | Natural.killer.T.cell | Innate |
| KLRG1 | Natural.killer.T.cell | Innate |
| HSPA4 | Natural.killer.T.cell | Innate |
| HSPB6 | Natural.killer.T.cell | Innate |
| ISM2 | Natural.killer.T.cell | Innate |
| ITIH2 | Natural.killer.T.cell | Innate |
| KDM4C | Natural.killer.T.cell | Innate |
| KIR2DS4 | Natural.killer.T.cell | Innate |
| KIRREL3 | Natural.killer.T.cell | Innate |
| SDCBP | Natural.killer.T.cell | Innate |
| NFATC2IP | Natural.killer.T.cell | Innate |
| MICB | Natural.killer.T.cell | Innate |
| KIR2DL1 | Natural.killer.T.cell | Innate |
| KIR2DL3 | Natural.killer.T.cell | Innate |
| KIR3DL1 | Natural.killer.T.cell | Innate |
| KIR3DL2 | Natural.killer.T.cell | Innate |
| NCR1 | Natural.killer.T.cell | Innate |
| FOSL1 | Natural.killer.T.cell | Innate |
| TSLP | Natural.killer.T.cell | Innate |
| SLC7A7 | Natural.killer.T.cell | Innate |
| SPP1 | Natural.killer.T.cell | Innate |
| TREM2 | Natural.killer.T.cell | Innate |
| UBASH3A | Natural.killer.T.cell | Innate |
| YBX2 | Natural.killer.T.cell | Innate |
| CCDC88A | Natural.killer.T.cell | Innate |
| CLEC1A | Natural.killer.T.cell | Innate |
| THBD | Natural.killer.T.cell | Innate |
| PDPN | Natural.killer.T.cell | Innate |
| VCAM1 | Natural.killer.T.cell | Innate |
| EMR1 | Natural.killer.T.cell | Innate |
| CREB5 | Neutrophil | Innate |
| CDA | Neutrophil | Innate |
| CHST15 | Neutrophil | Innate |
| S100A12 | Neutrophil | Innate |
| APOBEC3A | Neutrophil | Innate |
| CASP5 | Neutrophil | Innate |
| MMP25 | Neutrophil | Innate |
| HAL | Neutrophil | Innate |
| C1orf183 | Neutrophil | Innate |
| FFAR2 | Neutrophil | Innate |
| MAK | Neutrophil | Innate |
| CXCR1 | Neutrophil | Innate |
| STEAP4 | Neutrophil | Innate |
| MGAM | Neutrophil | Innate |
| BTNL8 | Neutrophil | Innate |
| CXCR2 | Neutrophil | Innate |
| TNFRSF10C | Neutrophil | Innate |
| VNN3 | Neutrophil | Innate |
| CBX6 | Plasmacytoid.dendritic.cell | Innate |
| DAB2 | Plasmacytoid.dendritic.cell | Innate |
| DDX17 | Plasmacytoid.dendritic.cell | Innate |
| HIGD1A | Plasmacytoid.dendritic.cell | Innate |
| IDH3A | Plasmacytoid.dendritic.cell | Innate |
| IL3RA | Plasmacytoid.dendritic.cell | Innate |
| MAGED1 | Plasmacytoid.dendritic.cell | Innate |
| NUCB2 | Plasmacytoid.dendritic.cell | Innate |
| OFD1 | Plasmacytoid.dendritic.cell | Innate |
| OGT | Plasmacytoid.dendritic.cell | Innate |
| PDIA4 | Plasmacytoid.dendritic.cell | Innate |
| SERTAD2 | Plasmacytoid.dendritic.cell | Innate |
| SIRPA | Plasmacytoid.dendritic.cell | Innate |
| TMED2 | Plasmacytoid.dendritic.cell | Innate |
| ENG | Plasmacytoid.dendritic.cell | Innate |
| FCAR | Plasmacytoid.dendritic.cell | Innate |
| IGF1 | Plasmacytoid.dendritic.cell | Innate |
| ITGA2B | Plasmacytoid.dendritic.cell | Innate |
| GABARAP | Plasmacytoid.dendritic.cell | Innate |
| GPX1 | Plasmacytoid.dendritic.cell | Innate |
| KRT23 | Plasmacytoid.dendritic.cell | Innate |
| PROK2 | Plasmacytoid.dendritic.cell | Innate |
| RALB | Plasmacytoid.dendritic.cell | Innate |
| RETNLB | Plasmacytoid.dendritic.cell | Innate |
| RNF141 | Plasmacytoid.dendritic.cell | Innate |
| SEC14L1 | Plasmacytoid.dendritic.cell | Innate |
| SEPX1 | Plasmacytoid.dendritic.cell | Innate |
| EMP3 | Plasmacytoid.dendritic.cell | Innate |
| CD300LF | Plasmacytoid.dendritic.cell | Innate |
| ABTB1 | Plasmacytoid.dendritic.cell | Innate |
| KLHL21 | Plasmacytoid.dendritic.cell | Innate |
| PHRF1 | Plasmacytoid.dendritic.cell | Innate |
|  |  |  |

| **Table S3** Immune signatures used in this study | |
| --- | --- |
| Metagene | Signatures |
| ARG1 | Immune suppression |
| ENTPD1 | Immune suppression |
| MICA | Immune suppression |
| MICB | Immune suppression |
| PDCD1LG2 | Immune suppression |
| TGFB1 | Immune suppression |
| VEGFA | Immune suppression |
| VEGFB | Immune suppression |
| KLRD1 | Cytolytic Activity |
| KLRF1 | Cytolytic Activity |
| GNLY | Cytolytic Activity |
| CTSW | Cytolytic Activity |
| KLRB1 | Cytolytic Activity |
| KLRK1 | Cytolytic Activity |
| NKG7 | Cytolytic Activity |
| GZMH | Cytolytic Activity |
| SIGIRR | Cytolytic Activity |
| ZBTB16 | Cytolytic Activity |
| RUNX3 | Cytolytic Activity |
| APOL3 | Cytolytic Activity |
| RORA | Cytolytic Activity |
| APBA2 | Cytolytic Activity |
| WHAMMP2 | Cytolytic Activity |
| DUSP2 | Cytolytic Activity |
| GZMA | Cytolytic Activity |
| B2M | Antigen processing machinery |
| HLA-A | Antigen processing machinery |
| HLA-B | Antigen processing machinery |
| HLA-C | Antigen processing machinery |
| TAP1 | Antigen processing machinery |
| TAP2 | Antigen processing machinery |
| HLA-A | Antigen processing machinery |
| HLA-B | Antigen processing machinery |
| HLA-C | Antigen processing machinery |
| HLA-DPA1 | Antigen processing machinery |
| HLA-DPB1 | Antigen processing machinery |
| HLA-DQA1 | Antigen processing machinery |
| HLA-DQA2 | Antigen processing machinery |
| HLA-DQB1 | Antigen processing machinery |
| HLA-DQB2 | Antigen processing machinery |
| HLA-DRA | Antigen processing machinery |
| HLA-DRB1 | Antigen processing machinery |
| HLA-DRB3 | Antigen processing machinery |
| HLA-DRB4 | Antigen processing machinery |
| HLA-DRB5 | Antigen processing machinery |
| MICA | Antigen processing machinery |
| MICB | Antigen processing machinery |
|  |  |
